# *PSAT1* positively regulates the osteogenic lineage differentiation of periodontal ligament stem cells through the ATF4/PSAT1/Akt/GSK3β/β-catenin axis

**DOI:** 10.1186/s12967-022-03775-z

**Published:** 2023-02-02

**Authors:** Linglu Jia, Dongfang Li, Ya-Nan Wang, Dongjiao Zhang, Xin Xu

**Affiliations:** 1 Department of Prosthodontics, School and Hospital of Stomatology, Cheeloo College of Medicine, Shandong University, Jinan, China; 2 Department of Implantology, School and Hospital of Stomatology, Cheeloo College of Medicine, Shandong University, No. 44-1 Wenhua Road West, Jinan, 250012 Shandong China; 3 Shandong Key Laboratory of Oral Tissue Regeneration, Jinan, China; 4 Shandong Engineering Laboratory for Dental Materials and Oral Tissue Regeneration, Jinan, China; 5 Shandong Provincial Clinical Research Center for Oral Diseases, Jinan, China

**Keywords:** PSAT1, Periodontal ligament stem cells, Osteogenic differentiation, Microarray assay

## Abstract

**Background:**

Periodontal ligament stem cells (PDLSCs) are important seed cells for tissue engineering to realize the regeneration of alveolar bone. Understanding the gene regulatory mechanisms of osteogenic lineage differentiation in PDLSCs will facilitate PDLSC-based bone regeneration. However, these regulatory molecular signals have not been clarified.

**Methods:**

To screen potential regulators of osteogenic differentiation, the gene expression profiles of undifferentiated and osteodifferentiated PDLSCs were compared by microarray and bioinformatics methods, and *PSAT1* was speculated to be involved in the gene regulation network of osteogenesis in PDLSCs. Lentiviral vectors were used to overexpress or knock down *PSAT1* in PDLSCs, and then the proliferation activity, migration ability, and osteogenic differentiation ability of PDLSCs in vitro were analysed. A rat mandibular defect model was built to analyse the regulatory effects of *PSAT1* on PDLSC-mediated bone regeneration in vivo. The regulation of *PSAT1* on the Akt/GSK3β/β-catenin signalling axis was analysed using the Akt phosphorylation inhibitor Ly294002 or agonist SC79. The potential sites on the promoter of *PSAT1* that could bind to the transcription factor ATF4 were predicted and verified.

**Results:**

The microarray assay showed that the expression levels of 499 genes in PDLSCs were altered significantly after osteogenic induction. Among these genes, the transcription level of *PSAT1* in osteodifferentiated PDLSCs was much lower than that in undifferentiated PDLSCs. Overexpressing *PSAT1* not only enhanced the proliferation and osteogenic differentiation abilities of PDLSCs in vitro, but also promoted PDLSC-based alveolar bone regeneration in vivo, while knocking down *PSAT1* had the opposite effects in PDLSCs. Mechanistic experiments suggested that *PSAT1* regulated the osteogenic lineage fate of PDLSCs through the Akt/GSK3β/β-catenin signalling axis. *PSAT1* expression in PDLSCs during osteogenic differentiation was controlled by transcription factor ATF4, which is realized by the combination of ATF4 and the *PSAT1* promoter.

**Conclusion:**

*PSAT1* is a potential important regulator of the osteogenic lineage differentiation of PDLSCs through the ATF4/PSAT1/Akt/GSK3β/β-catenin signalling pathway. *PSAT1* could be a candidate gene modification target for enhancing PDLSCs-based bone regeneration.

**Supplementary Information:**

The online version contains supplementary material available at 10.1186/s12967-022-03775-z.

## Introduction

Periodontal disease, trauma, tumours, etc. can cause the destruction and loss of alveolar bones, damaging the physiological function of people. Rapidly developing bone tissue engineering has become an attractive approach to achieve the regeneration of defective bones [1, 2]. Seed cells, scaffolds and growth factors, which are the main component elements of bone tissue engineering, have been widely studied and show beneficial effects on bone regeneration [3].

Periodontal ligament stem cells (PDLSCs), a type of adult mesenchymal stem cell (MSC), have received increasing attention as seed cells for bone tissue engineering, because they have considerable capabilities in proliferation, osteogenic lineage differentiation and anti-apoptosis [4–7]. In addition, the tissue sources of PDLSCs are rich and minimally invasive, because they can be obtained from extracted third molars [5]. Several studies have proven that the use of culture-expanded PDLSCs with various biomaterials or growth factors promoted the regeneration of defective maxillofacial bone to a certain degree in animal models [8–11]. However, there is still a gap between the effects of tissue engineering and clinical expectations.

Elaborating the molecular mechanisms underlying the osteogenic differentiation of PDLSCs will help to explore methods to enhance PDLSC-based bone regeneration. To date, a large number of genes and proteins, and the signalling pathways in which they participate have been proven to affect the osteogenic differentiation of PDLSCs, including RUNX2 [12], Wnt/β-catenin signalling pathway-related proteins [13], PI3K/Akt signalling pathway-related proteins [14], MAPK signalling pathway-related proteins [15], etc. However, the exact inner regulatory networks that underlie the osteogenic lineage fate of PDLSCs remain unclear. To search for potential important regulators of osteogenic differentiation, the present study filtered differentially expressed coding-genes between undifferentiated and osteodifferentiated PDLSCs by microarray assay, and conducted a primary analysis through bioinformatics methods. Notably, *PSAT1*, the expression of which was altered significantly during the osteogenic differentiation of PDLSCs, seemed to be a potential regulator.

Phosphoserine aminotransferase 1 (PSAT1), encoded by the gene *PSAT1*, is a type of protein with enzyme activity that participates in l-serine metabolism [16]. *PSAT1* is widely expressed in tissue cells and has been proven to affect a variety of physiological functions. For example, hepatic *Psat1* was demonstrated to regulate insulin sensitivity in mice [17]. *PSAT1* was reported to be required for collagen protein production by lung fibroblasts in humans [18]. Mutations in the *PSAT1* gene lead to the development of some mental diseases and congenital developmental abnormalities [19–22]. In addition, *PSAT1* was reported to be overexpressed in some types of tumours and could affect the proliferation or invasion of cancer cells, including esophageal squamous cell carcinoma, colon cancer, and non-small cell lung cancer cells [23–27]. It is worth noting that a recent study proved that *Psat1* affected the timing of mouse embryonic stem cell (ESC) differentiation in a serine-independent manner [28]. Another study revealed a connection between *Psat1*expression and extracellular matrix mineralization in mouse osteoblasts [29]. In addition, the serine metabolic pathway that *PSAT1* involved in was reported to affect the ageing of dental pulp stem cells [30]. Based on the above facts and the results of the microarray assay, we speculated that *PSAT1* may play a regulatory role in the osteogenic differentiation of PDLSCs and aimed to investigate this possibility.

In this study, we provided evidence of the regulatory effects of *PSAT1* on PDLSC osteogenic differentiation in vivo and in vitro. In addition, we revealed that *PSAT1* affected the osteogenesis of PDLSCs through the Akt/GSK3β/β-catenin signalling pathway. Furthermore, *PSAT1*expression was regulated by the transcription factor ATF4 during the osteogenic differentiation of PDLSCs. We hope that these results provide new ideas for the study of gene regulatory networks of osteogenic lineage differentiation in PDLSCs.

## Materials and methods

### Cell isolation and cultivation

Healthy premolars that were extracted due to orthodontic treatment were collected. The donors were aged from 16 to 24 years-old and were systemically healthy. The periodontal ligament tissues were scraped from the middle third of the root surface and minced. Then, the tissues were attached to the bottom of the culture flask and incubated for 2 h at 37 °C in a 5% CO_2_ incubator. Finally, the complete culture medium containing alpha-minimal essential medium (α-MEM) (BI, Beit Haemek, Israel) and 10% foetal bovine serum (FBS) (BI) was added to the culture flask. The medium was refreshed every 3 days after the cells grew out of the tissue masses. Cells were passaged using trypsin/EDTA (Solarbio, Beijing, China) after reaching 90% confluence, and cells at passages 3–6 were used for the experiments.

### Immuno-phenotype assay

To analyse the immune-phenotype of PDLSCs, the BD Human MSC Analysis Kit (BD Biosciences, NJ, USA) was used according to the manufacturer’s instructions. In brief, cells were harvested and incubated with antibodies conjugated with fluorescent dyes in the dark at 4 ℃ for 30 min. Then, the cells were washed with phosphate-buffered saline (PBS) and detected by flow cytometry. Antibodies of the kit included MSC-positive markers (CD90, CD105, CD73, CD44) and MSC-negative markers (CD34, CD11, CD19, CD45, HLA-DR).

### Clonogenic assay

PDLSCs were seeded into 6-well culture plates at a density of 200 cells per well and cultured in complete culture medium for 10 days. Then, the cells were fixed with paraformaldehyde and stained with 0.1% crystal violet (Solarbio). Finally, the cells were observed under the microscope, and aggregates with more than 50 cells were regarded as clones.

### Multilineage differentiation assay

For the osteogenic differentiation assay, PDLSCs were cultured in osteogenic medium containing α-MEM (BI), 10% FBS (BI), 10 nM dexamethasone (Solarbio), 10 mM β-glycerophosphate (Solarbio) and 50 mg/L ascorbic acid (Solarbio). After 4 weeks, the cells were fixed with paraformaldehyde and stained with Alizarin Red solution (Sigma, St. Louis, MO, USA) to detect mineralized nodules. To analyse the intensity of Alizarin red staining, mineralized nodules were dissolved in 10% cetylpyridinium chloride (Solarbio) and quantified using a microplate reader at 562 nm. When necessary, Ly294002 (#9901, CST) (an inhibitor of Akt phosphorylation) or SC79 (HY-18749, MCE, USA) (a promoter of Akt phosphorylation) was added to the osteogenic medium at concentration of 10 µmol/L or 5 µg/mL, respectively.

For the adipogenic differentiation assay, PDLSCs were cultured in adipogenic medium containing α-MEM (BI), 10% FBS (BI), 1 µM dexamethasone (Solarbio), 0.2 mM indomethacin (Solarbio), 0.01 g/L insulin (Solarbio) and 0.5 mM isobutyl-methylxanthine (Solarbio). Four weeks later, the cells were fixed with paraformaldehyde and stained with oil red O (Solarbio) to detect lipid droplets.

### Microarray analyses and bioinformatics analyses

Three PDLSC clones obtained from three different individuals were cultured in osteogenic medium for 7 days (OI PDLSCs), and the corresponding PDLSCs cultured in complete culture medium for 7 days were regarded as the control (NC PDLSCs). Then, the cells were collected for microarray analyses through GeneChip Human Transcriptome Array 2.0 (Affymetrix, USA) according to the manufacturer’s instructions with the help of a company (GMINIX Informatics Ltd., Co., Shanghai, China). The differentially expressed genes between OI PDLSCs and NC PDLSCs were filtered using the significance analysis of microarrays (SAM) method [31]. Fold change (FC), which reflected the change in gene expression level, was calculated. and its absolute value represented the change range, and the positive or negative sign represented upregulation or downregulation.

The differentially expressed genes were further analysed through GO analyses and pathway analyses. GO analyses were based on the Gene Ontology Consortium database, which identified the function of genes in terms of biological process (BP), cellular component (CC) and molecular function (MF). Pathway analyses were based on the Kyoto Encyclopedia of Genes and Genomes (KEGG) database, which confirmed which biological pathways these differentially expressed genes participated in. Furthermore, hybrid hierarchical clustering algorithm was used to analyse the relationships among differentially expressed genes, and the correlation coefficient of each pair was calculated.

### Cell proliferation assay

The cell counting Kit-8 kit (CCK-8) (Dojindo Laboratories, Kumamoto, Japan) and EdU detection kit (RiboBio, Guangzhou, China) were used to analyse cell proliferation according to the manufacturer’s instructions. For the CCK-8 assay, cells were incubated in complete culture medium containing 10% CCK-8 solution at 37 ℃ for 2 h, and then the absorbance at 450 nm was measured by a microplate reader. For the EdU assay, cells were incubated in 50 µM EdU labelling medium at 37 ℃ for 2 h, and then fixed and stained with Apollo® 567 solution and Hoechst 33342 solution. Afterwards, the cells were observed under a fluorescence microscope and photographed. The percentage of EdU-positive cells among the total cells was calculated based on 6 random fields.

### Cell cycle assay

To detect the cell cycle distribution of cells, a cell cycle and apoptosis analysis kit (Beyotime, Shanghai, China) was used according to the manufacturer’s instructions, and then the cells were analysed by flow cytometry.

### ALP staining and quantitative analysis of ALP activity

To measure the activity of alkaline phosphatase (ALP), ALP staining and quantitative analysis of ALP activity were used. For ALP staining, PDLSCs were stained using the NBT/BCIP staining kit (Beyotime) according to the manufacturer’s instructions, and then the cells were observed under microscope. For the quantitative analysis of ALP activity, PDLSCs were lysed and assayed using an ALP assay kit (Nanjing Jiancheng Bioengineering Institute, Nanjing, China).

### Cell migration assay

Cells were plated in a culture dish with complete culture medium. At 90% confluency, a vertical line was scratched in the culture dish using a sterile pipette tip, and the exfoliated cells were washed off by PBS. The cells in the culture dish were incubated in α-MEM (BI) supplemented with 0.5% FBS (BI), and images at the 0th hour, 24th hour, and 36th hour were captured under a microscope. Finally, ImageJ software was used to analyse the migration areas.

### Small interfering RNA (siRNA) and plasmid transfection

siRNAs were designed and synthesized by GenePharma Corporation (Shanghai, China) to downregulate the expression of target genes, and siRNA-targeted none was used as the control (siNC group). The siRNA primers are shown in Additional file 1. To overexpress *ATF4*, the pcDNA3.1 vector containing full-length *ATF4* was used (OE ATF4 group), and the empty pcDNA3.1 vector was used as the control (pcDNA3.1 group). Transfection of PDLSCs with siRNAs and plasmids was performed using Micropoly-transfecter cell reagent (Micropoly, Jiangsu, China) according to the manufacturer’s protocol.

### Lentivirus transfection

To overexpress or knockdown *PSAT1* in PDLSCs, the *PSAT1*-overexpression vector (pGLV3H1-GFP-puro) and the short hairpin RNA (shRNA) duplex oligo targeting *PSAT1* (pEF-1αF-GFP-puro) were packaged into lentiviruses (Genechem, Shanghai, China). The pGLV3H1-GFP-puro empty vector and nontargeting shRNA pEF-1αF-GFP-puro were also packaged into lentiviruses as a control. PDLSCs were incubated in complete culture medium supplemented with lentiviruses and polybrene (Genechem) for 6 h, and then incubated in complete culture medium supplemented with puromycin (Solarbio) for 1 week. Finally, PDLSCs with *PSAT1* overexpression (OEPSAT1), PDLSCs with *PSAT1* knockdown (shPSAT1), and their respective control groups (OENC, shNC) were obtained.

### Protein extraction and Western Blot

The total proteins of cells were extracted using RIPA buffer (Solarbio) containing protease inhibitor (Solarbio) and phosphatase inhibitor (Bosterbio, Wuhan, China). The cytoplasmic and nuclear proteins of cells were extracted using the Nuclear and Cytoplasmic Protein Extraction Kit (Bosterbio). The proteins were separated by SDS-PAGE and transferred to polyvinylidene difluoride membranes. Then, the membranes were blocked with nonfat milk and incubated with primary antibodies overnight at 4 °C, followed by incubation with secondary antibodies conjugated with horseradish peroxidase. Finally, the signals on the membranes were detected by enhanced chemiluminescence reagent under an Amersham Imager 600 (General Electric Company, USA), and the grey values of the protein bands were analysed by ImageJ software.

Primary antibodies included the following: PSAT1 (ab96136, Abcam, Cambridge, MA, USA); ALP (ab108337, Abcam); COL1A1 (#84336, CST, Danvers, MA, USA); RUNX2 (ab23981, Abcam); Akt (pan) (#4691, CST); p-Akt (Ser473) (p-Akt) (#4060, CST); p-GSK3β (#9323, CST); GSK-3β (#12456, CST); β-Catenin (#8480, CST); Nonphospho (Active) β-Catenin (#8814, CST); ATF4 (#11815, CST); β-Actin (sc-517582, CST); Histone-H3 (17168-1-AP, Proteintech, Chicago, IL, USA); and GAPDH (HRP-60,004, Proteintech).

### RNA isolation and quantitative real-time polymerase chain reaction (qRT-PCR)

Total RNA was extracted from cells using Total RNA Extraction Reagent (Vazyme, Nanjing, China) according to the manufacturer’s instructions. Then, the RNA was reverse transcribed to cDNA using HiScript III RT SuperMix for qPCR (+gDNA wiper) (Vazyme). qRT-PCR was performed in a 10 µL reaction volume with ChamQ Universal SYBR qPCR Master Mix (Vazyme), and the changes in gene expression were calculated by the 2^−ΔΔCT^ method. GAPDH was used as the internal control. The primers of the genes are shown in Additional file 2.

### Construction of the rat mandibular defect model

Seven-week-old male Wistar rats (200–250 g) supplied by SPF Biotechnology Co., Ltd. (Beijing, China) were kept in the animal centre of Shandong Engineering Laboratory for Dental Materials and Oral Tissue Regeneration. After 1 week of acclimation, the rats were anaesthetized by the intraperitoneal injection of pentobarbital sodium anaesthesia (40 mg/kg body weight). An incision parallel to the inferior border of the mandible was made, the skins and muscles were separated, and a defect (5 × 3 × 1 mm^3^) was made on the buccal surface of the mandible using a dental round bur at a low speed with continuous physiological saline solution irrigation. The anterior margin of the defect was 2 mm from the anterior edge of the mandible, and the lower margin was located 1 mm above the lower edge of the mandible. Then, PDLSC-bone powder mixtures from different groups were implanted into the defect and covered with biomembranes (Zhenghai Biotechnology, China) to prevent the growth of soft tissue. Finally, muscles and subcutaneous tissues were sutured layer by layer with resorbable sutures. Penicillin sodium (160,000 IU/mL) was injected intramuscularly during the first 3 days after the surgery, and a soft diet was provided for 1 week. Each group had 4 rats, and the rats were sacrificed by cardiac perfusion at 8 weeks after the surgery. Then, the mandibular specimens were harvested. The mandibular specimens were fixed in 4% paraformaldehyde for 48 h, and then prepared for microscopic computerized tomography (micro-CT) analysis, histomorphometric analysis and Masson’s trichrome staining analysis.

For the preparation of the PDLSC-bone powder mixture, 4 × 10^6^ OEPSAT1 PDLSCs, OENC PDLSCs, shPSAT1 PDLSCs or shNC PDLSCs were cultured in osteogenic medium for 7 days. Then, the cells were mixed with 6 mg of bone powder (BioOss, Geistlich Biomaterials, Switzerland) and incubated in a 5% CO_2_ incubator at 37 °C for 6 h before surgery.

### Micro-CT analysis

The mandibular specimens were scanned using a micro-CT scanner (µCT-100, SCANCO Medical AG, Switzerland) with an effective pixel size of 15 μm. Then, the three-dimensional images were reconstructed, and the volumetric parameters of the newly formed bone in the surgical area were analysed, including the bone volume per tissue volume (BV/TV, %), the trabecular bone thickness (Tb.-Th, mm), the trabecular bone separation (Tb.-Sp, mm), and the total number of trabeculae (Tb.N; mm).

### Haematoxylin–eosin (HE) staining and Masson’s trichrome staining

The mandibular specimens were decalcified in 10% disodium ethylenediamine tetraacetate in phosphate buffer for approximately 2 months, dehydrated through a graded series of ethanol solutions and embedded in paraffin. The specimens were cut to generate series sections (5-µm thickness) from the mesial to distal direction, which involved the whole surgical area. Respective sections of the defects were used for HE staining and Masson’s trichrome staining with the Hematoxylin–Eosin/HE Staining Kit (Solarbio) and the Masson’s Trichrome Stain Kit (Solarbio).

### Chromatin immunoprecipitation (ChIP) assay

ChIP experiments were performed using the ChIP Assay Kit (Beyotime) according to the manufacturer’s instructions. An antibody against ATF4 (#11815, CST) was applied, and rabbit IgG (A7016, Beyotime) was used as a control. The ChIP products were amplified by qRT-PCR. The primers for qRT-PCR were as follows: forward primer (5′–3′) GGCGCATCAATTTTACTCAGAC; reverse primer (5′–3′) CAGAATACCCTCCCCCTACCC.

### Statistical analysis

All experiments were repeated at least three times, and all data are presented as the means ± standard deviations. Student’s t test or one-way ANOVA was used to determine significant differences between experimental and control values. The statistical analyses were conducted using GraphPad software, and values of *p* < 0.05 were considered significant.

## Results

### Culture and identification of PDLSCs

**Fig. 1:**
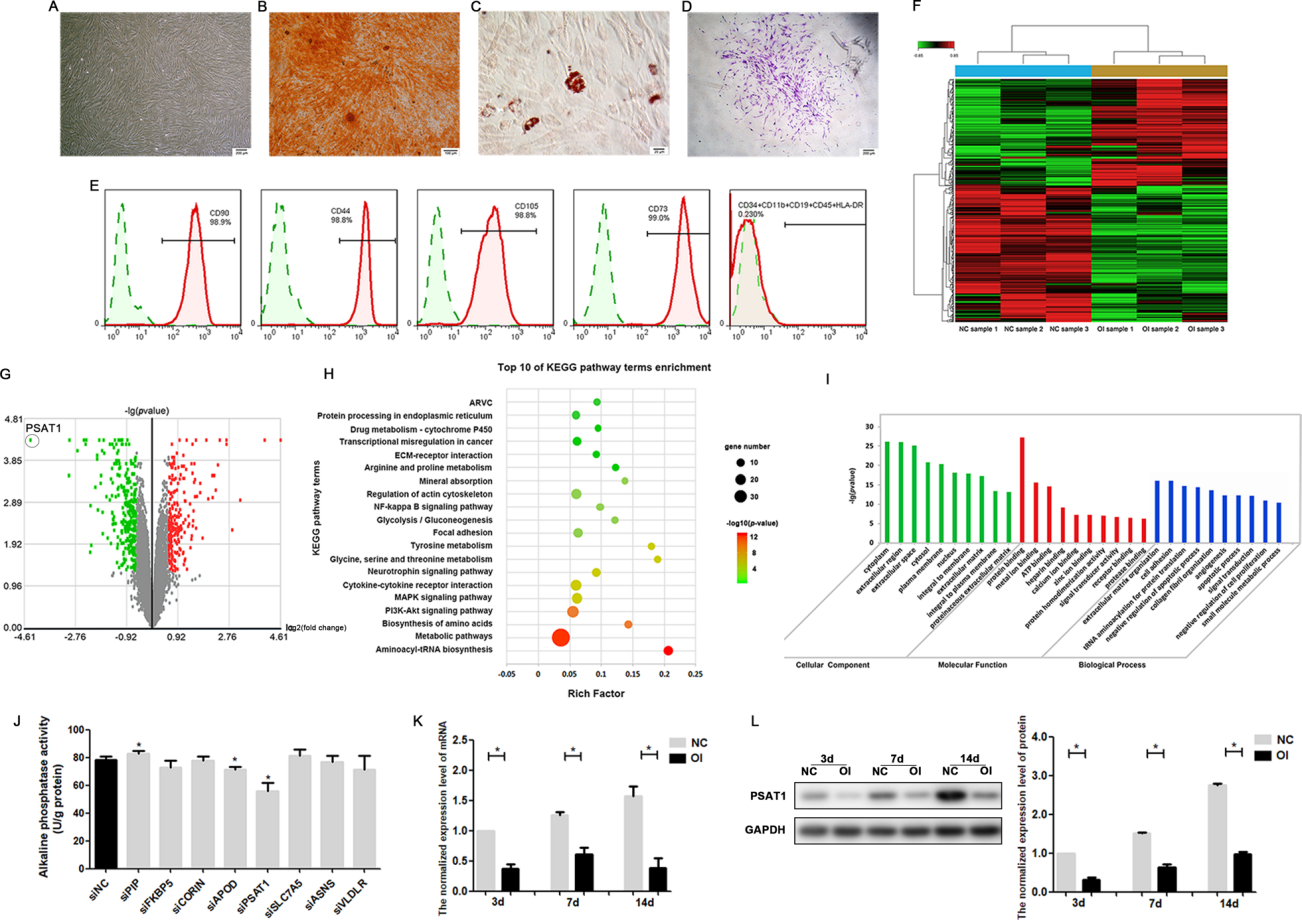
Microarray and bioinformatics analyses for differentially expressed genes in PDLSCs after osteogenic induction. **A** PDLSCs cultured in vitro presented spindle-like morphology and formed spiral arrangements. **B** Alizarin Red staining of PDLSCs after osteogenic induction for 4 weeks. **C** Oil Red O staining of PDLSCs after adipogenic induction for 4 weeks. **D** Clonogenic assay of PDLSCs. **E** Immunophenotype assay of PDLSCs. **F** Clustering heatmap of differentially expressed mRNAs in PDLSCs after osteogenic induction for 7 days. **G** Volcano plot of differentially expressed mRNAs in PDLSCs after osteogenic induction for 7 days. **H** The top 10 KEGG pathways enriched by differentially expressed mRNAs in PDLSCs. **I** The top 10 GO terms enriched by differentially expressed mRNAs in PDLSCs. **J** The quantitative analysis of ALP activity in PDLSCs after siRNAs were transfected. **K** The mRNA level of *PSAT1* in PDLSCs after osteogenic induction for 3, 7, and 14 days. **L** The protein level of PSAT1 in PDLSCs after osteogenic induction for 3, 7, and 14 days. NC: PDLSCs that were cultured in the complete culture medium. OI: PDLSCs that were cultured in the osteogenic medium. *p < 0.05

PDLSCs cultured in vitro exhibited spindle-like morphology and formed spiral arrangements (Fig. 1A). In the multilineage differentiation assay, PDLSCs produced Alizarin Red-positive mineralized matrix (Fig. 1B) or Oil Red O-positive lipid droplets (Fig. 1C) after being cultured in osteogenic or adipogenic medium for 4 weeks, which confirmed that PDLSCs possessed the ability of osteogenic and adipogenic differentiation. In the clonogenic assay, PDLSCs showed strong proliferative ability, and each cell could form a colony composed of more than 50 cells (Fig. 1D). In the immunophenotype assay, PDLSCs positively expressed mesenchymal cell specific surface markers such as CD73, CD90, CD105 and CD44, and negatively expressed haematopoietic or endothelial-specific antigens including CD34, CD11b, CD19, CD45 and HLA-DR (Fig. 1E). All above results proved that the PDLSCs obtained in this experiment were consistent with the characteristics of MSCs.

### Microarray and bioinformatics analyses for differentially expressed genes in PDLSCs after osteogenic induction

To identify potential regulators of osteogenic differentiation in PDLSCs, we used a GeneChip microarray assay to compare the mRNA expression profiles between osteodifferentiated PDLSCs and control PDLSCs. According to the requirements of *p* < 0.05 and the absolute value of FC ≥ 1.5, the expression levels of 499 genes in PDLSCs were screened to change significantly after osteogenic induction, including 219 upregulated and 280 downregulated (Fig. 1F, G, Additional file 3). To verify the accuracy of the microarray assay, qRT-PCR was used to further detect the expression level of these screened genes. The results of qRT-PCR on 6 representative genes were highly consistent with those of microarray data, suggesting that the microarray assay was reliable (Additional file 4).

GO analyses and pathway analyses were performed to understand the function of differentially expressed genes in PDLSCs. In GO analyses, all of the differentially expressed genes participated in 326 BP terms, 50 CC terms and 88 MF terms. According to the *p* value, the top 10 GO terms are shown in Fig. 1I. In pathway analyses, all of the differentially expressed genes were enriched in 89 significant signalling pathways, and the top 10 terms according to *p* value are shown in Fig. 1H, which showed that pathways such as metabolic pathways, the PI3K-Akt signalling pathway, the MAPK signalling pathway, and cytokine‒cytokine receptor interaction pathway could play regulatory roles in the osteogenic lineage differentiation of PDLSCs.

Based on all of the above results, the screened differentially expressed genes with high FC were thought to be more likely to be related to the osteogenic differentiation of PDLSCs. To further screen genes that play important roles, siRNAs were used to inhibit the expression of 8 representative genes with high FC (*PIP*, *FKBP5*, *CORIN*, *APOD*, *PSAT1*, *SLC7A5*, *ASNS*, and *VLDLR*) in PDLSCs during osteogenic induction (Additional file 5). As shown in Fig. 1J, the ALP activity of PDLSCs decreased significantly after *PSAT1* was knocked down, suggesting that *PSAT1* may participate in the regulation of osteogenic differentiation. We detected *PSAT1* mRNA and PSAT1 protein on the 3rd, 7th and 14th day of osteogenic induction, and found that *PSAT1* expression in osteodifferentiated PDLSCs was always lower than that in undifferentiated PDLSCs. Based on all of the above experiments, we speculated that *PSAT1* participated in the regulation of osteogenic differentiation in PDLSCs, and thus carried out more experiments to verify this hypothesis.

### Overexpression and knockdown efficiency of *PSAT1* in PDLSCs

**Fig. 2:**
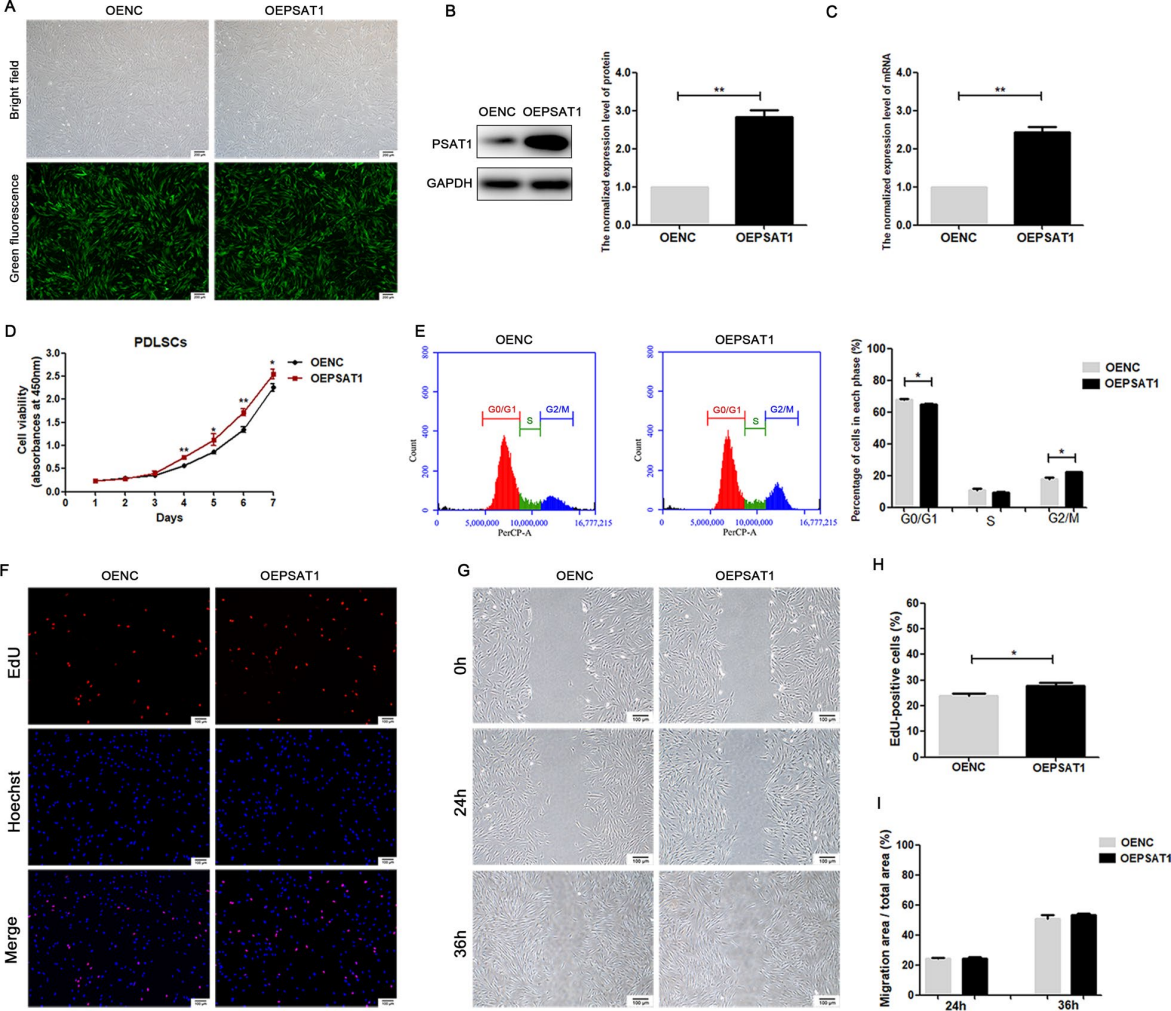
The effect of *PSAT1* overexpression on proliferation activity and migration ability of PDLSCs.  **A** PDLSCs transfected with lentivirus were observed under fluorescence microscope. **B**, **C** The expression levels of PSAT1 protein and *PSAT1* mRNA in PDLSCs were detected by Western Blot and qRT-PCR. **D** Cell viability of PDLSCs was detected by CCK-8. **E** The distribution of the cell cycle in PDLSCs was detected by flow cytometry. **F**, **H** Cell proliferation activity of PDLSCs was detected by EdU, and the proportion of EdU-positive cells was calculated. **G**, **I** Migration ability of PDLSCs was analyzed, and the migration efficiency was calculated. OEPSAT1: PDLSCs with *PSAT1* overexpressed. OENC: control PDLSCs. *p < 0.05; **p < 0.01

**Fig. 3:**
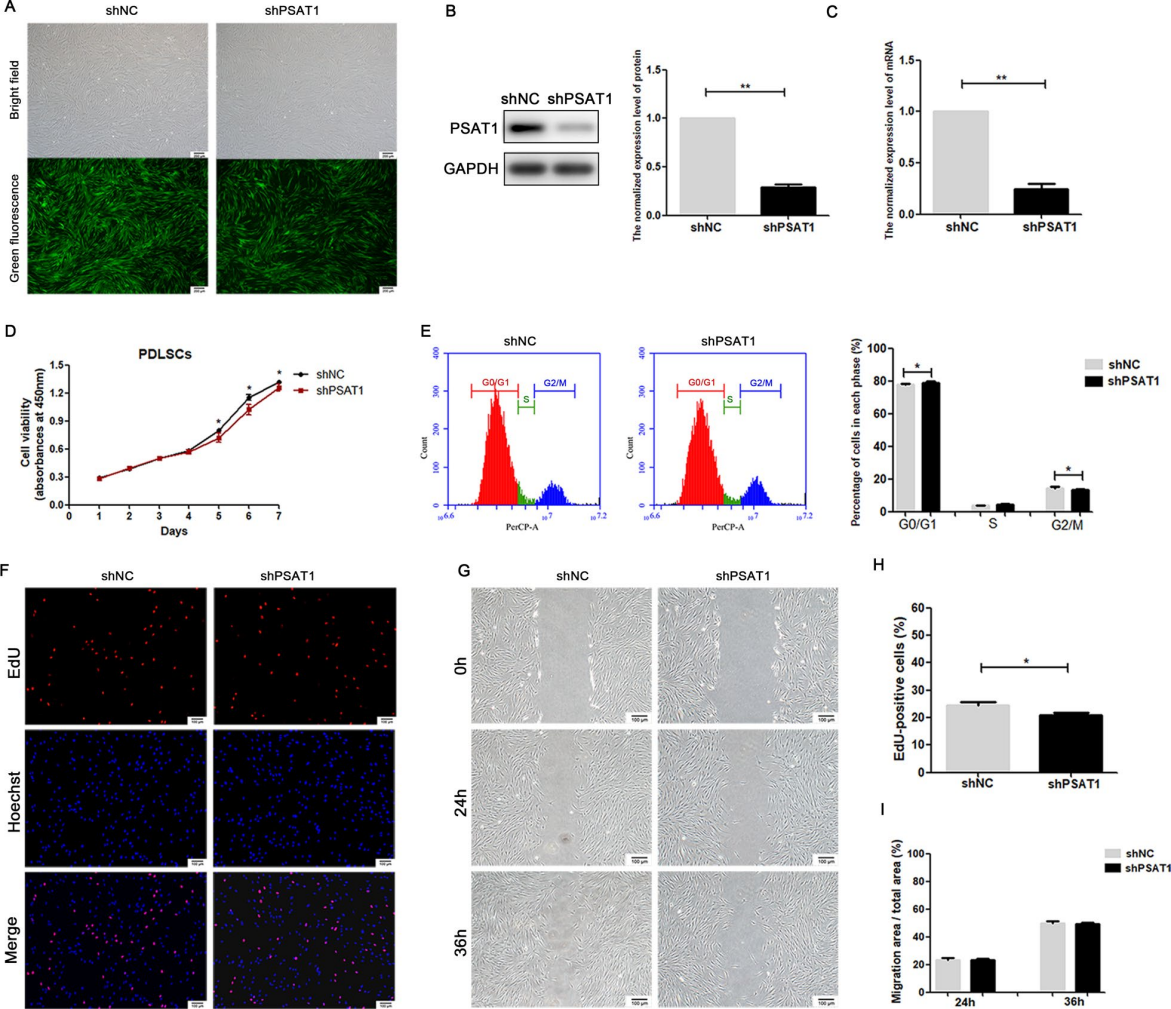
The effect of ***PSAT1*** knockdown on proliferation activity and migration ability of PDLSCs. **A** PDLSCs transfected with lentivirus were observed under fluorescence microscope. **B**, **C** The expression levels of PSAT1 protein and *PSAT1* mRNA in PDLSCs were detected by Western Blot and qRT-PCR. **D** Cell viability of PDLSCs was detected by CCK-8. **E** The distribution of the cell cycle in PDLSCs was detected by flow cytometry. **F**, **H** Cell proliferation activity of PDLSCs was detected by EdU, and the proportion of EdU-positive cells was calculated. **G**, **I** Migration ability of PDLSCs was analyzed, and the migration efficiency was calculated. shPSAT1: PDLSCs with *PSAT1* knocked down. shNC: control PDLSCs. *p < 0.05; **p < 0.01

Lentiviral vectors with green fluorescent labels were used to overexpress or knock down *PSAT1* expression in PDLSCs. As observed under a fluorescence microscope, more than 90% of PDLSCs were successfully transfected with lentivirus (Figs. 2A, 3A). Next, the mRNA and protein expression levels of *PSAT1* in PDLSCs were measured by qRT-PCR and Western Blot. The results showed that the mRNA and protein levels of *PSAT1* were higher in OEPSAT1 PDLSCs than in OENC PDLSCs (Fig. 2B, C), while those in shPSAT1 PDLSCs were lower than those in shNC PDLSCs (Fig. 3B, C), indicating that *PSAT1* was successfully overexpressed or knocked down in PDLSCs. These cells were used in the following study.

### *PSAT1* regulated PDLSC proliferation in vitro

Cell proliferation activity has a great influence on PDLSC-based bone regeneration; thus, CCK-8, EdU, and cell cycle flow cytometric analyses were performed to analyse the effects of overexpressing and knocking down *PSAT1* on PDLSC proliferation.

When *PSAT1* was overexpressed, the CCK-8 assay on 7-day growth curves showed that the cell viability of the OEPSAT1 group was significantly higher than that of the OENC group after 4 days (Fig. 2D). In the cell cycle distribution assay, the percentage of cells in G2/M phase in the OEPSAT1 group was higher than that in the OENC group (Fig. 2E). In the EdU assay, PDLSCs were cultured for 4 days before EdU labelling, and the percentage of EdU-positive cells, which are in the active stage of DNA replication, was higher in the OEPSAT1 group than in the OENC group (Fig. 2F, H). All of the above results indicated that overexpressing *PSAT1* promoted PDLSC.

When *PSAT1* was knocked down, shPSAT1 PDLSCs had lower cell viability than shNC PDLSCs after culturing for 5 days (Fig. 3D), the percentage of cells in G2/M phase in the shPSAT1 group was lower than that in the shNC group (Fig. 3E), and the EdU-positive rates of shPSAT1 PDLSCs were lower than those of shNC PDLSCs (Fig. 3F, H). These results suggested that knocking down *PSAT1* impaired the proliferative activity of PDLSCs.

### *PSAT1* did not affect the migration ability of PDLSCs in vitro

The migration ability of seed cells is of great significance to tissue regeneration. To detect whether *PSAT1* affected the migration ability of PDLSCs, the migration area of PDLSCs was measured at 24 and 36 h after scratching. The results showed no significant difference in cell migration areas between the OENC group and the OEPSAT1 group at the above two time points (Fig. 2G, I). When *PSAT1* was knocked down, the migration areas of cells in the shPSAT1 group and the shNC group were similar (Fig. 3G, I). Thus, overexpression or knockdown of *PSAT1* had no significant effect on PDLSC migration.

### *PSAT1* regulated the osteogenic differentiation of PDLSCs in vitro

To study the regulation effects of *PSAT1* on the osteogenic differentiation ability of PDLSCs, OENC, OEPSAT1, shNC and shPSAT1 PDLSCs were cultured in osteogenic medium for 21 days.

**Fig. 4:**
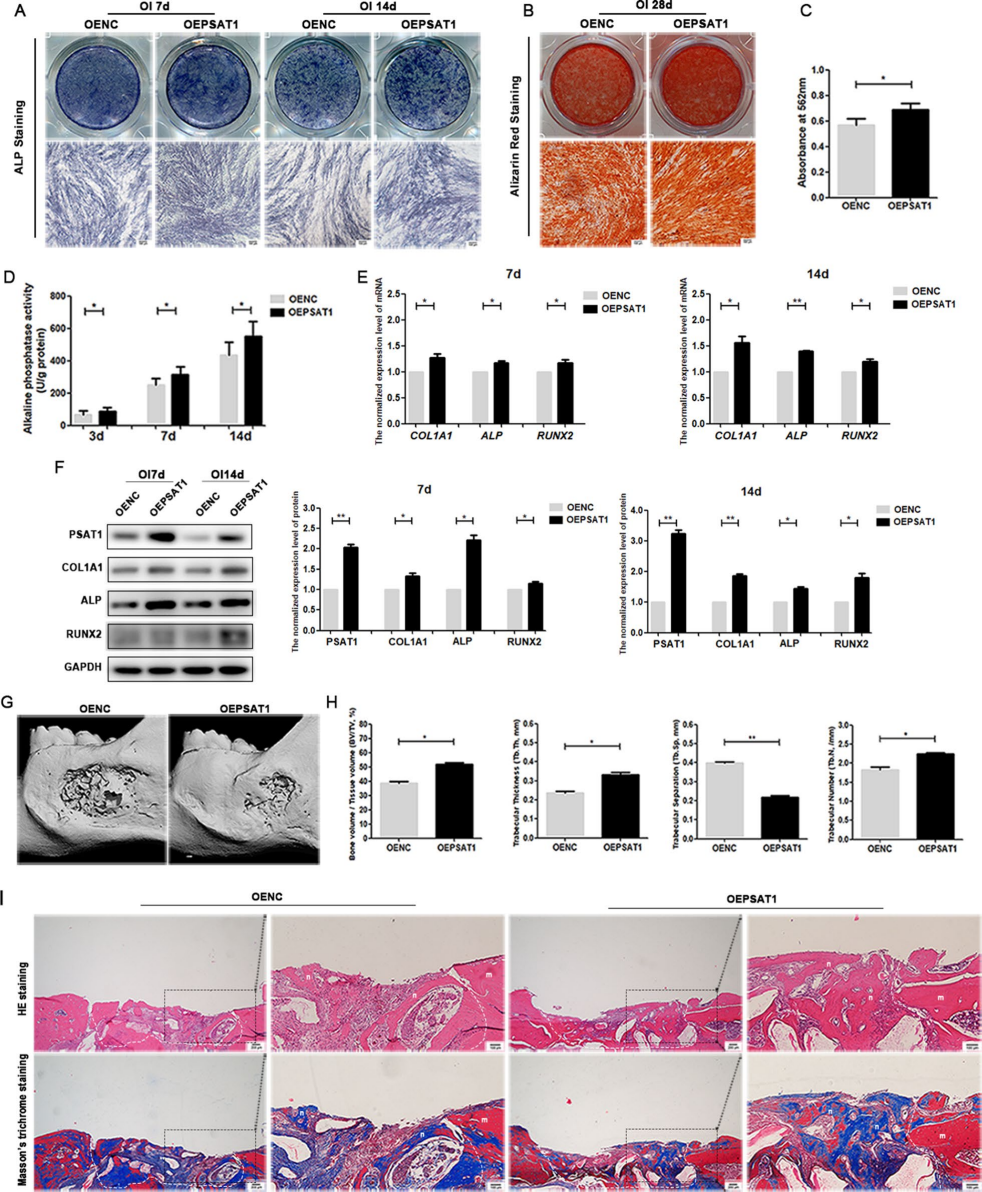
The effect of *PSAT1* overexpression on osteogenic differentiation of PDLSCs in vitro and bone regeneration in vivo. **A** ALP staining of PDLSCs after osteogenic induction for 7 and 14 days. **B** Alizarin red staining of PDLSCs after osteogenic induction for 21 days. **C** Quantitative analysis of mineralized matrix after osteogenic induction for 21 days. **D** The quantitative analysis of ALP activity in PDLSCs after osteogenic induction. **E** The mRNA levels of *COL1A1*, *ALP* and *RUNX2* in PDLSCs after osteogenic induction. **F** The protein levels of COL1A1, ALP and RUNX2 in PDLSCs after osteogenic induction. **G**, **H** Micro-CT assay of rat mandibular defect areas was performed after PDLSCs were transplanted, and the bone volume per tissue volume (BV/TV, %), the trabecular bone thickness (Tb. Th, mm), the trabecular bone separation (Tb.Sp, mm), and the total number of trabeculae (Tb.N; mm) were calculated. **I** HE staining and Masson’s trichrome staining of rat mandibular defect areas. White dotted line: bone defect boundary, n: new bone, f: collagen fiber, m: jaw bone. OEPSAT1: PDLSCs with *PSAT1* overexpressed. OENC: control PDLSCs. *p < 0.05; **p < 0.01

When comparing OENC PDLSCs and OEPSAT1 PDLSCs, the ALP activity of OEPSAT1 PDLSCs on Days 3, 7, and 14 was stronger than that of OENC PDLSCs (Fig. 4D). Correspondingly, the ALP staining of the OEPSAT1 group on Days 7 and 14 was also deeper than that of the OENC group (Fig. 4A). The results of Alizarin red staining and quantitative analysis showed that OEPSAT1 PDLSCs formed more mineralized matrix than OENC PDLSCs after osteogenic induction for 21 days (Fig. 4B, C). The expression of osteogenic differentiation markers such as *ALP*, *COL1A1* and *RUNX2* was also detected, and the results showed that the protein and mRNA levels of these markers in OEPSAT1 PDLSCs were higher than those in OENC PDLSCs (Fig. 4E, F). Thus, *PSAT1* overexpression enhanced the osteogenic differentiation ability of PDLSCs.

**Fig. 5:**
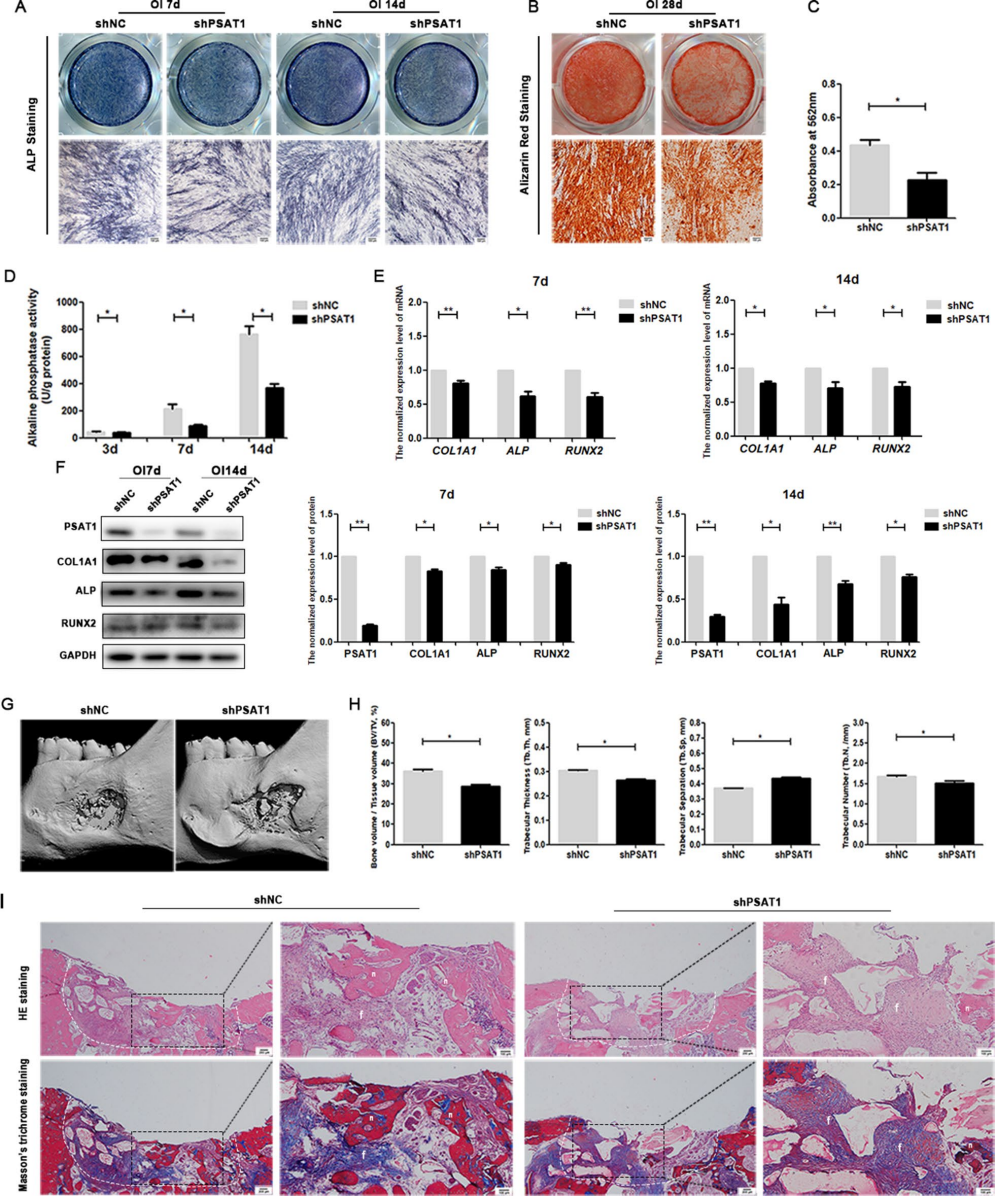
The effect of *PSAT1* knockdown on osteogenic differentiation of PDLSCs in vitro and bone regeneration in vivo. **A** ALP staining of PDLSCs after osteogenic induction for 7 and 14 days. **B** Alizarin red staining of PDLSCs after osteogenic induction for 21 days. **C** Quantitative analysis of mineralized matrix after osteogenic induction for 21 days. **D** The quantitative analysis of ALP activity in PDLSCs after osteogenic induction. **E** The mRNA levels of *COL1A1*, *ALP* and *RUNX2* in PDLSCs after osteogenic induction. **F** The protein levels of COL1A1, ALP and RUNX2 in PDLSCs after osteogenic induction. **G**, **H** Micro-CT assay of rat mandibular defect areas was performed after PDLSCs were transplanted, and the bone volume per tissue volume (BV/TV, %), the trabecular bone thickness (Tb. Th, mm), the trabecular bone separation (Tb.Sp, mm), and the total number of trabeculae (Tb.N; mm) were calculated. **I** HE staining and Masson’s trichrome staining of rat mandibular defect areas. White dotted line: bone defect boundary, n: new bone, f: collagen fiber, m: jaw bone. shPSAT1: PDLSCs with *PSAT1* knocked down. shNC: control PDLSCs. *p < 0.05; **p < 0.01

When *PSAT1* was knocked down, the ALP activity of shPSAT1 PDLSCs was weaker than that of shNC PDLSCs (Fig. 5D). The ALP staining of shPSAT1 PDLSCs was also lighter than that of shNC PDLSCs (Fig. 5A). Less mineralized matrix was formed in the shPSAT1 group than in the shNC group (Fig. 5B, C). In addition, the protein and mRNA expression of *ALP*, *COL1A1*, and *RUNX2* in shPSAT1 PDLSCs was lower than that in shNC PDLSCs (Fig. 5E, F). These results indicated that knocking down *PSAT1* inhibited the osteogenic differentiation of PDLSCs.

### *PSAT1* influenced PDLSC-mediated bone regeneration in vivo

To investigate the influence of *PSAT1* on PDLSC-mediated bone regeneration, PDLSCs with *PSAT1* overexpression or knockdown were transplanted into rat mandibular defect areas, and then micro-CT, HE staining and Masson’s trichrome staining were used to analyze the healing of the defects and evaluate the quantity and quality of newly formed bone.

When *PSAT1* was overexpressed, the micro-CT results at 8 weeks showed that the OEPSAT1 group had a higher BV/TV value, a higher Tb.-Th value, a higher Tb.-N value, and a lower Tb.-Sp value than the OENC group (Fig. 4G, H). In the HE staining assay, more newly formed bones were observed in the OEPSAT1 group than in the OENC group (Fig. 4I). In the Masson’s trichrome staining assay, more newly formed bones, which were rich in collagen type I stained blue, were detected in the OEPSAT1 group than in the OENC group (Fig. 4I). All of the above results suggested that overexpressing *PSAT1* improved PDLSC-based bone regeneration in the mandibular defect model.

When comparing the shNC and shPSAT1 groups, a decrease in the BV/TV value, Tb.-Th value, and Tb.-N value, and an increase in Tb.-Sp values were observed in the shPSAT1 group in the micro-CT assay (Fig. 5G, H). The results of HE staining showed that newly formed bones were less abundant in the shPSAT1 group than in the shNC group (Fig. 5I). In the Masson’s trichrome staining assay, fewer new bones were formed in the shPSAT1 group than in the shNC group, which was consistent with the results of HE staining (Fig. 5I). All of these results indicated that knocking down *PSAT1* impaired PDLSC-based bone regeneration in mandibular defect area.

### *PSAT1* regulated the osteogenic differentiation of PDLSCs through the Akt/GSK3β/β-catenin signalling pathway

**Fig. 6:**
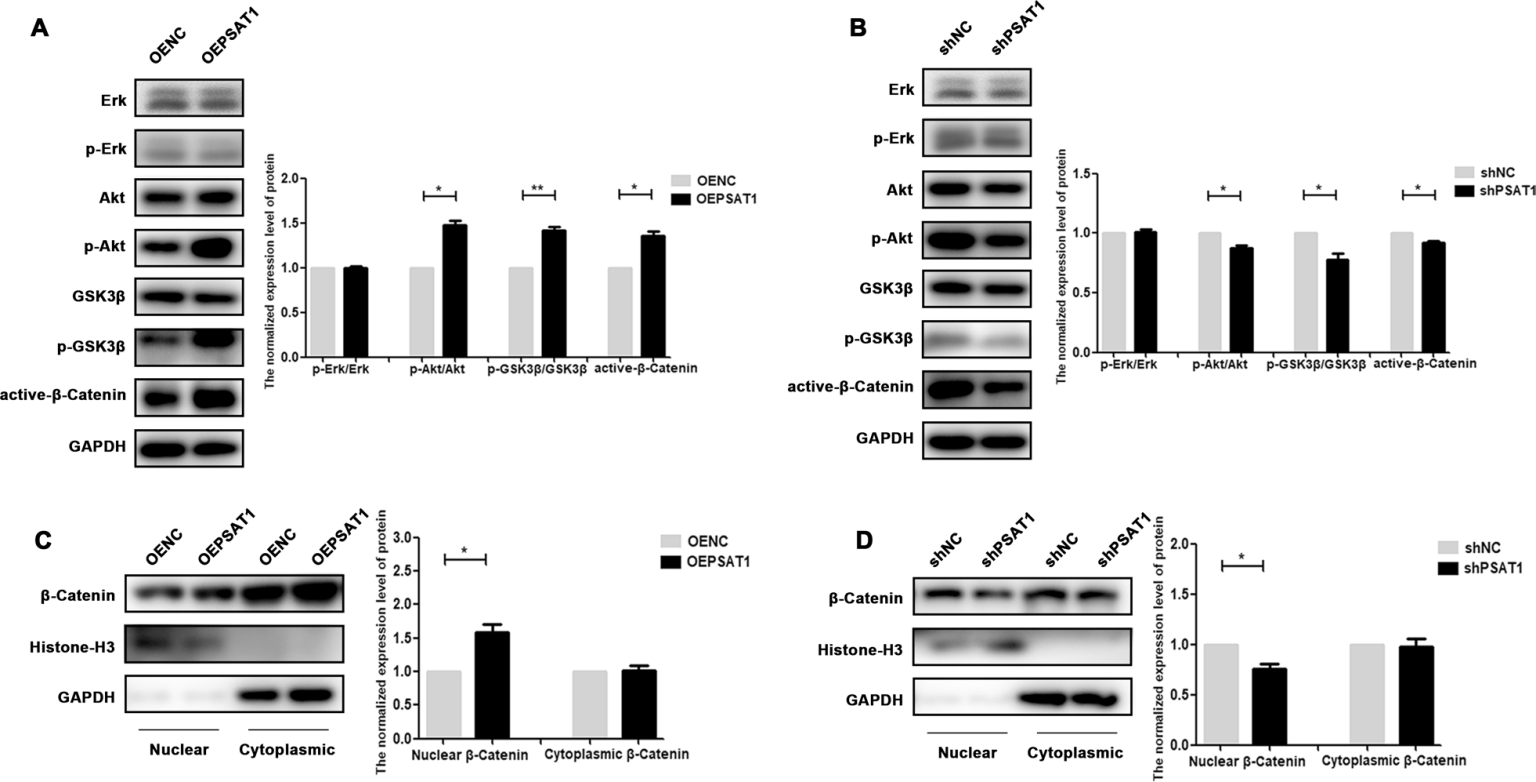
*PSAT1* regulated Akt/GSK3β/β-catenin signaling pathway in PDLSCs. **A** The phosphorylation level of Erk, Akt, and GSK3β, and the protein level of active-β-catenin were analyzed in PDLSCs with *PSAT1* overexpressed. **B** The phosphorylation level of Erk, Akt, and GSK3β, and the protein level of active-β-catenin were analyzed in PDLSCs with *PSAT1* knocked down. **C** Western Blot analysis of β-Catenin protein in nucleus and cytoplasm after *PSAT1* was overexpressed. **D** Western Blot analysis of β-Catenin protein in nucleus and cytoplasm after *PSAT1* was knocked down. OEPSAT1: PDLSCs with *PSAT1* overexpressed. OENC: control PDLSCs for OEPSAT1 PDLSCs. shPSAT1: PDLSCs with *PSAT1* knocked down. shNC: control PDLSCs for shPSAT1 PDLSCs. *p < 0.05; **p < 0.01

As mentioned above, the differentially expressed genes in PDLSCs after osteogenic induction were enriched in different signalling pathways, including the MAPK signalling pathway, and the PI3K/Akt signalling pathway. To explore the potential regulatory mechanisms of *PSAT1*, the phosphorylation of Erk and Akt, which reflect the activity of MAPK/Erk signalling and PI3K/Akt signalling respectively, was detected. As shown in Fig. 6A, B, the phosphorylation level of Erk in PDLSCs did not change significantly when *PSAT1* was overexpressed or knocked down. When the phosphorylation level of Akt was detected, it increased or decreased with the overexpression or knockdown of *PSAT1* (Fig. 6A, B). We further detected the expression of downstream factors of Akt signalling, including GSK3β and β-catenin. The Western Blot results showed that the phosphorylation level of GSK3β and the protein level of active-β-catenin (nonphospho β-catenin) in PDLSCs increased when *PSAT1* was overexpressed (Fig. 6A), while they decreased when *PSAT1* was knocked down (Fig. 6B). Consistent with the above results, the amount of β-catenin protein in the cell nucleus increased due to the overexpression of *PSAT1* (Fig. 6C), while it decreased when *PSAT1* was knocked down (Fig. 6D). All of the above results suggested that *PSAT1* could promote the conduction of Akt/GSK3β/β-catenin signals in PDLSCs.

**Fig. 7:**
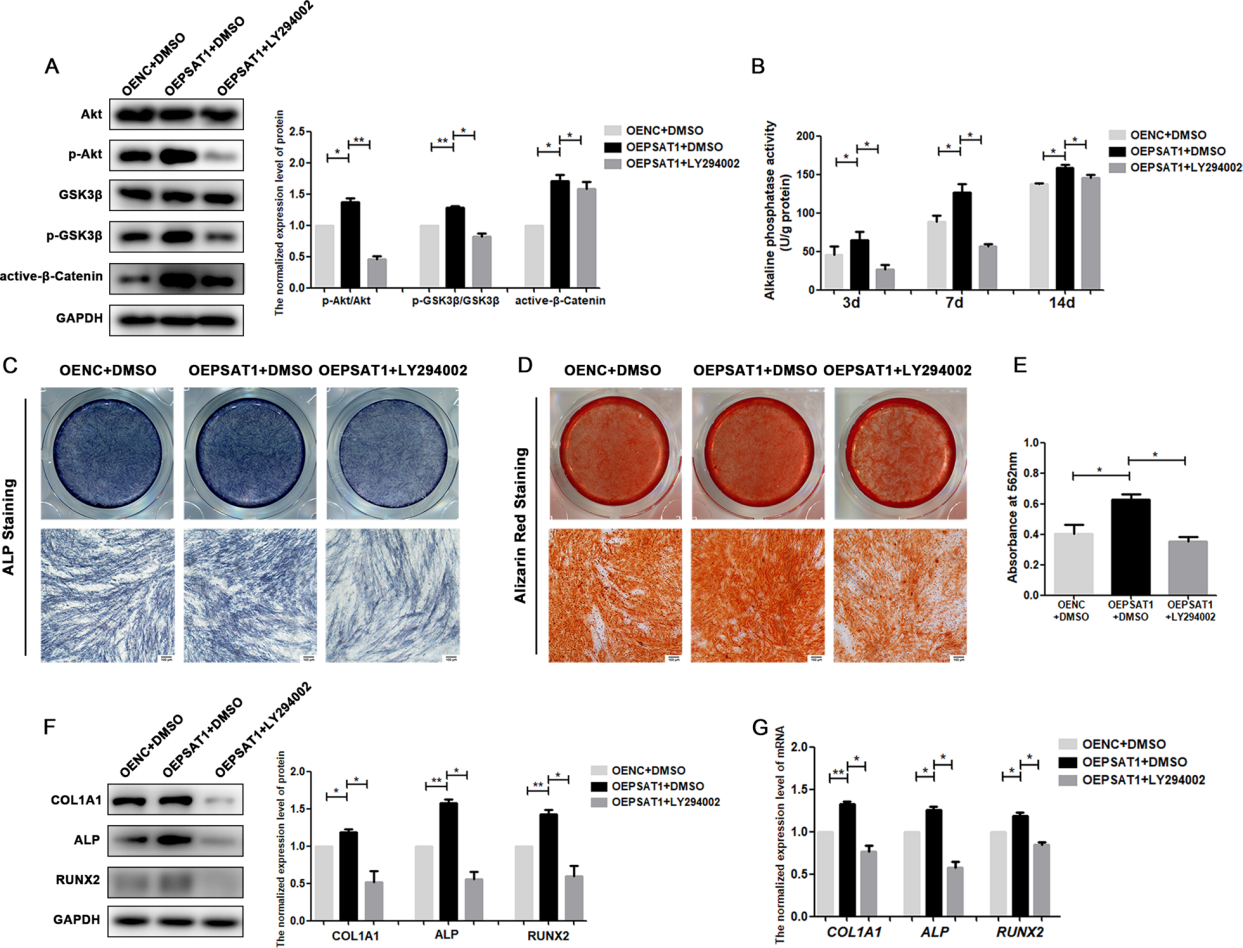
LY294002 reversed the effects of overexpressing *PSAT1* on the osteogenic differentiation of PDLSCs. **A** The phosphorylation level of Akt and GSK3β, and the protein level of active-β-catenin were analyzed in PDLSCs. **B** The quantitative analysis of ALP activity in PDLSCs after osteogenic induction. **C** ALP staining of PDLSCs after osteogenic induction for 7 days. **D** Alizarin red staining of PDLSCs after osteogenic induction for 21 days. **E** Quantitative analysis of mineralized matrix after osteogenic induction for 21 days. **F** The protein levels of COL1A1, ALP and RUNX2 in PDLSCs after osteogenic induction for 7 days. **G** The mRNA levels of *COL1A1*, *ALP* and *RUNX2* in PDLSCs after osteogenic induction for 7 days. OEPSAT1: PDLSCs with *PSAT1* overexpressed. OENC: control PDLSCs. *p < 0.05; **p < 0.01

**Fig. 8:**
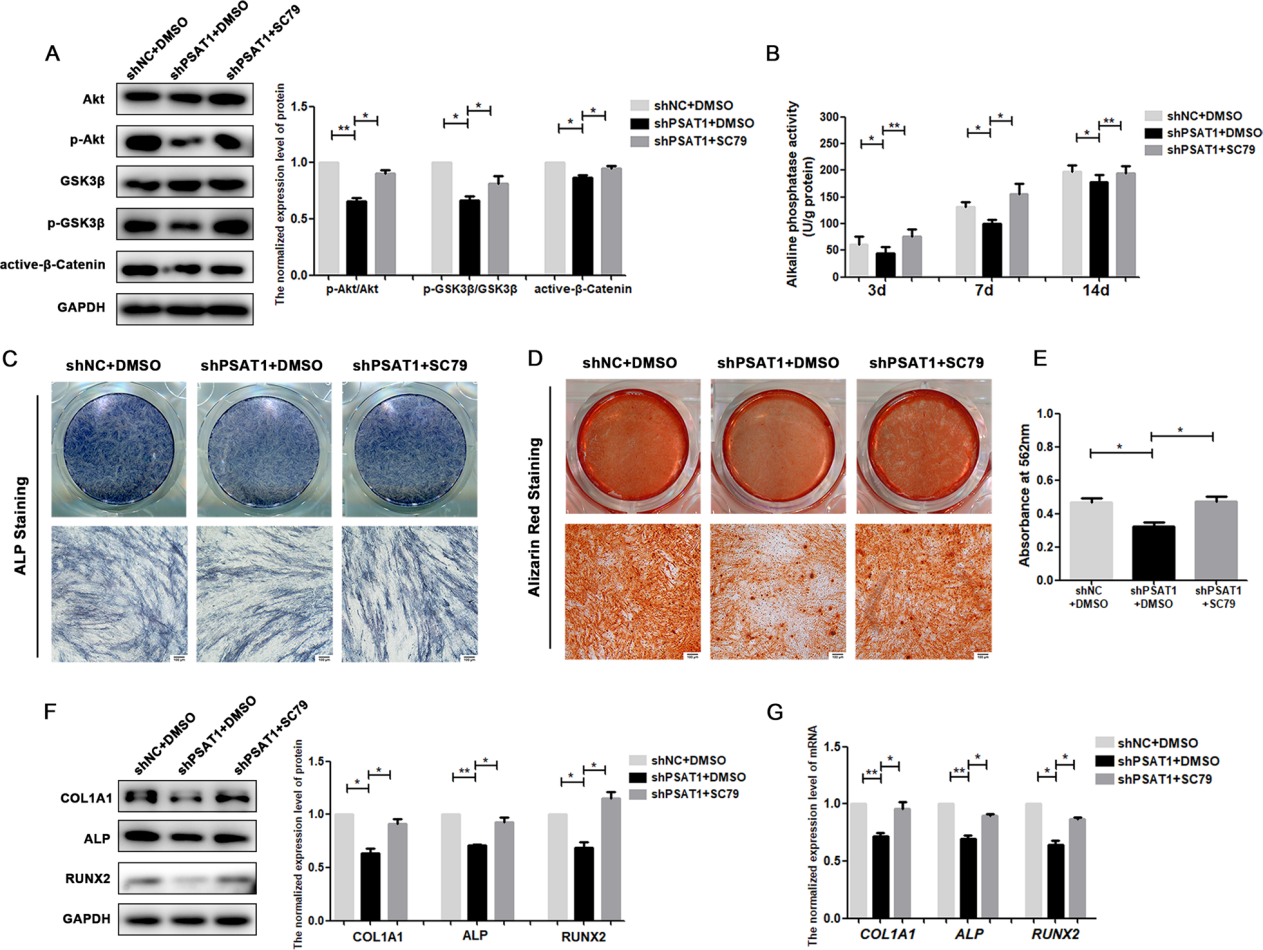
SC79 reversed the effects of knocking down *PSAT1* on the osteogenic differentiation of PDLSCs. **A** The phosphorylation level of Akt and GSK3β, and the protein level of active-β-catenin were analyzed in PDLSCs. **B** The quantitative analysis of ALP activity in PDLSCs after osteogenic induction. **C** ALP staining of PDLSCs after osteogenic induction for 7 days. **D** Alizarin red staining of PDLSCs after osteogenic induction for 21 days. **E** Quantitative analysis of mineralized matrix after osteogenic induction for 21 days. **F** The protein levels of COL1A1, ALP and RUNX2 in PDLSCs after osteogenic induction for 7 days. **G** The mRNA levels of *COL1A1*, *ALP* and *RUNX2* in PDLSCs after osteogenic induction for 7 days. shPSAT1: PDLSCs with *PSAT1* knocked down. shNC: control PDLSCs. *p < 0.05; **p < 0.01

To further validate whether *PSAT1* regulated the osteogenic differentiation of PDLSCs through the Akt/GSK3β/β-catenin signalling pathway, LY294002 or SC79, which can inhibit or promote the phosphorylation of Akt, was applied during the osteogenic induction of PDLSCs. As shown in Fig. 7A, the phosphorylation of Akt in the OEPSAT1 group was higher than that in the OENC group, but when LY294002 was added to the medium of the OEPSAT1 group, the higher phosphorylation level of Akt in OEPSAT1 PDLSCs was reduced (Fig. 7A). In the following experiments, LY294002 led to a decrease in ALP activity (Fig. 7B), ALP staining (Fig. 7C), mineralization of extracellular matrix (Fig. 7D, E), and expression of osteogenic differentiation markers (Fig. 7F, G) in the OEPSAT1 plus LY294002 group compared to the OEPSAT1 group, which suggested that inhibiting the phosphorylation of Akt could reverse the enhancement of osteogenic differentiation caused by overexpressing *PSAT1*. In addition, compared with the shPSAT1 group, the phosphorylation of Akt in shPSAT1 plus SC79 group increased (Fig. 8A), and stronger ALP activity (Fig. 8B, C), more mineralization of the extracellular matrix (Fig. 8D, E), and higher expression levels of osteogenic differentiation markers (Fig. 8F, G) were observed in the shPSAT1 plus SC79 group, which indicated that improving the phosphorylation level of Akt could reverse the decrease in osteogenic differentiation caused by the knockdown of *PSAT1*. All of the above results proved that overexpressing or knocking down *PSAT1* could regulate the osteogenic differentiation of PDLSCs through the Akt/GSK3β/β-catenin signalling pathway.

### *PSAT1* expression was regulated by the transcription factor ATF4

**Fig. 9:**
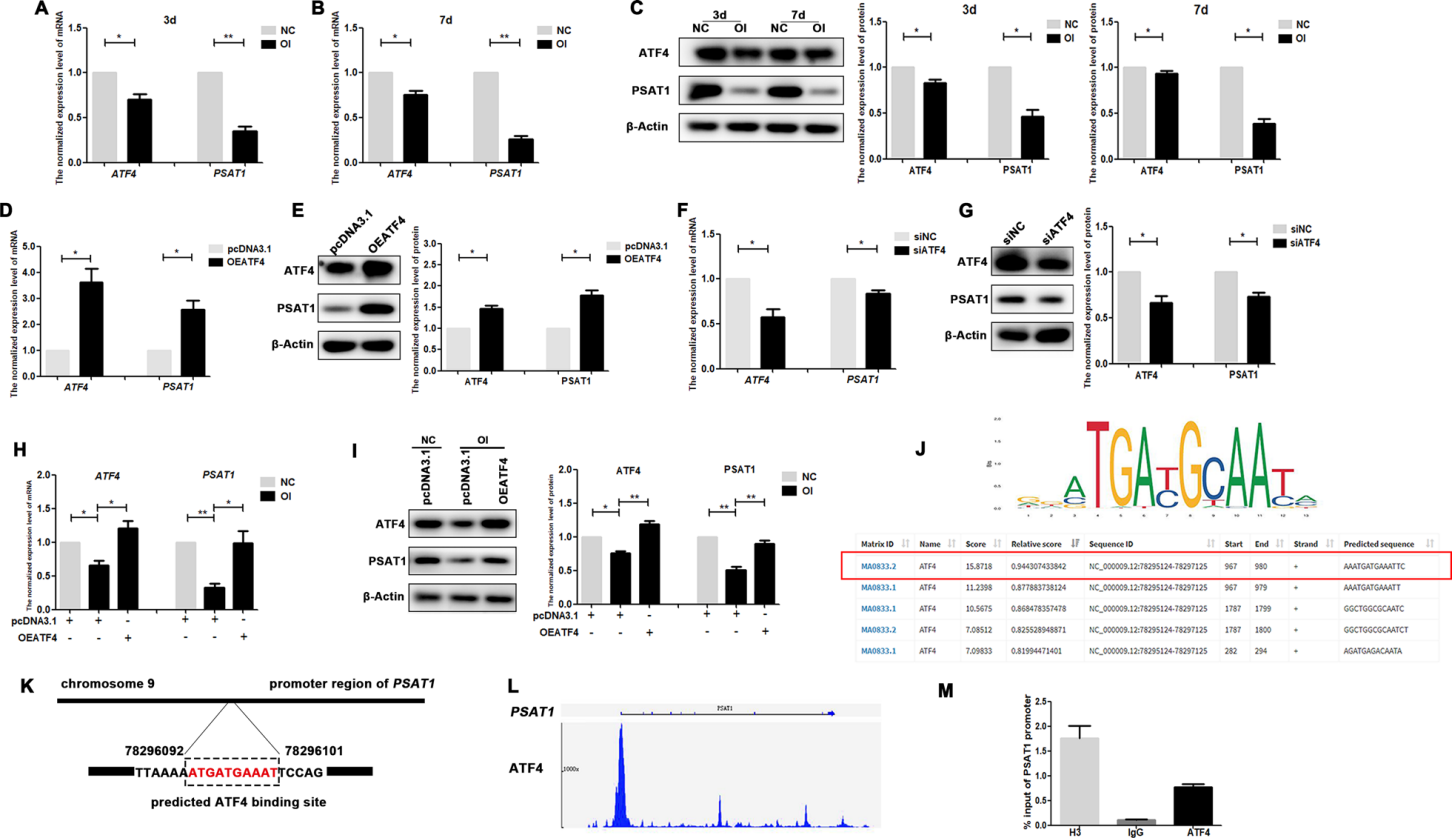
The expression of *PSAT1* was regulated by transcription factor ATF4. **A**, **B** The mRNA levels of *ATF4* and *PSAT1* in PDLSCs after osteogenic induction for 3 and 7 days. **C** The protein levels of ATF4 and PSAT1 in PDLSCs after osteogenic induction for 3 and 7 days. **D**, **E** The mRNA and protein levels of *ATF4* and *PSAT1* in PDLSCs after *ATF4* was overexpressed. **F**, **G** The mRNA and protein levels of *ATF4* and *PSAT1* in PDLSCs after *ATF4* was interfered. **H**, **I** The mRNA and protein levels of *ATF4* and *PSAT1* in PDLSCs with *ATF4* overexpressed after osteogenic induction for 7 days. **J** Jasper database and PROMO database were used to predict a combination of ATF4 protein and *PSAT1* promoter. **K** The predicted binding sites in promoter region of *PSAT1* that could bind to transcription factor ATF4. **L** GTRD database was used to analyze the combination peak of ATF4 and *PSAT1* promoter regions. **M** ChIP experiment verified that ATF4 could bind to the predicted region of *PSAT1*. NC: PDLSCs that were cultured in the complete culture medium. OI: PDLSCs that were cultured in the osteogenic medium. OEATF4: PDLSCs that were transfected with the pcDNA3.1 vector containing full-length *ATF4*. pcDNA3.1: PDLSCs that were transfected with empty pcDNA3.1 vector. siATF4: PDLSCs that were transfected with siRNA-targeted *ATF4*. siNC: PDLSCs that were transfected with siRNA-targeted none. *p < 0.05; **p < 0.01

To further clarify the signal network mediated by *PSAT1* in PDLSCs, we analysed the results of the microarray assay and screened 11 genes had coexpression relationships with *PSAT1* (Additional file 6). Among these genes, *ATF4*, which encodes a well-known transcription factor that can regulate the biological behaviour of stem cells, had a high correlation coefficient with *PSAT1* according to the microarray assay. To explore the connection between *ATF4* and *PSAT1*, the expression levels of *PSAT1* and *ATF4* in undifferentiated PDLSCs and osteodifferentiated PDLSCs were detected by qRT-PCR and Western Blot. As shown in Fig. 9A–C, the protein and mRNA levels of *ATF4* and *PSAT1* in PDLSCs showed a similar change trend after osteogenic induction for 3 and 7 days. Next, we used a plasmid or siRNA to upregulate or inhibit *ATF4* expression in PDLSCs, and found that the mRNA and protein levels of *PSAT1* increased or decreased when *ATF4* was overexpressed or knocked down (Fig. 9D–G). In addition, *ATF4* overexpression reversed the decrease in *PSAT1* expression levels after osteogenic induction (Fig. 9H, I). All of these results suggested a positive correlation between the expression of *PSAT1* and *ATF4.*

**Fig. 10:**
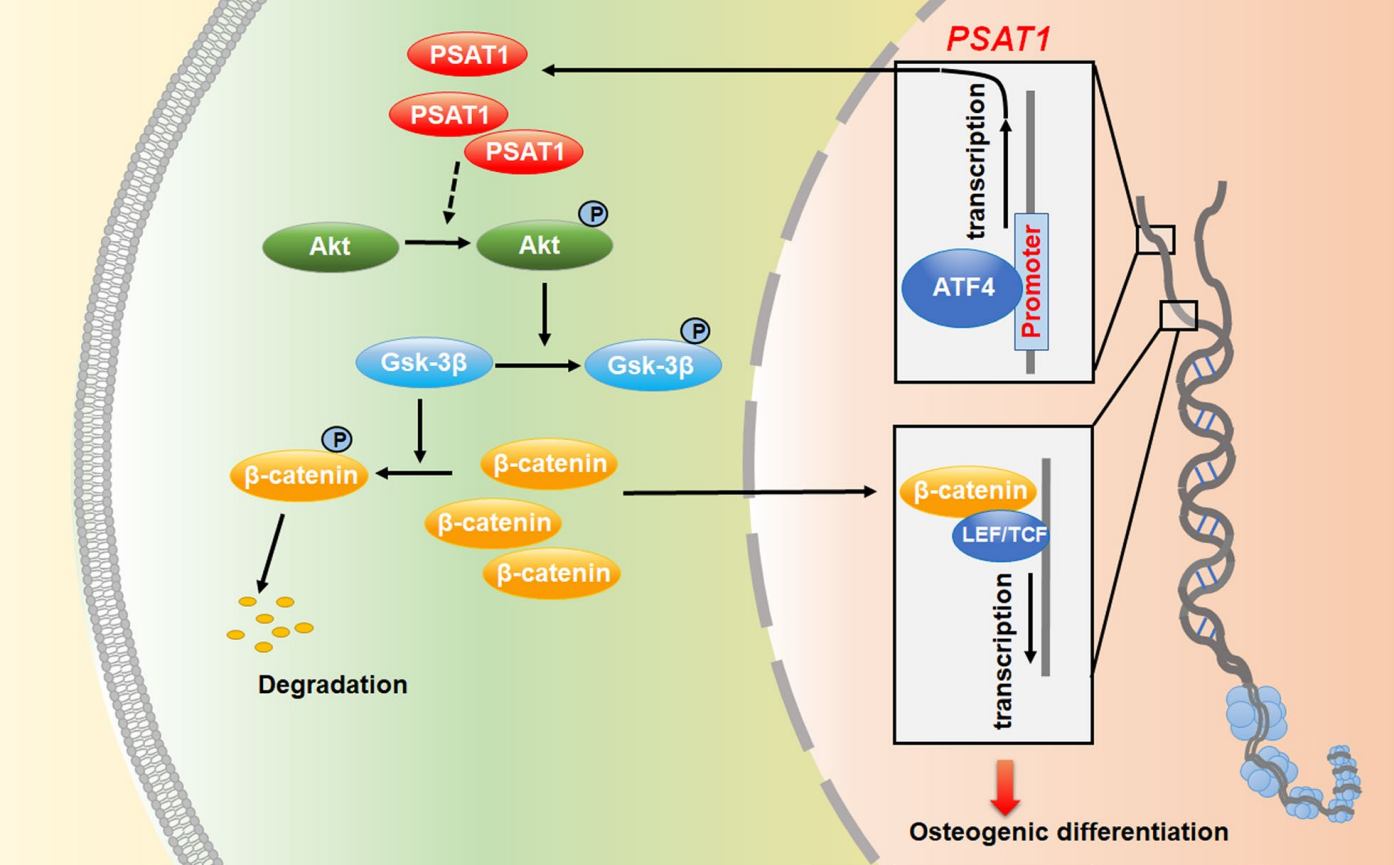
Schematic diagram. *PSAT1* could regulate the osteogenic differentiation of PDLSCs through Akt/GSK3β/β-catenin signaling pathway, and the transcription of *PSAT1* is modulated by transcription factor ATF4

Then, based on the assay of the Jasper database (http://jaspar.genereg.net/), PROMO database (http://alggen.lsi.upc.es/), and GTRD database (http://www.gtrd.com/), a binding site of transcription factor ATF4 was predicted in the promoter region of *PSAT1* (Fig. 9J–L), suggesting that transcription factor ATF4 could probably regulate the transcription of *PSAT1*. Finally, we performed ChIP experiments, and the results showed that transcription factor ATF4 could bind to the predicted site in the promoter region of *PSAT1* (Fig. 9M). All of the above results proved that *PSAT1* expression was regulated by ATF4 in PDLSCs, which regulated the ATF4/PSAT1/Akt/GSK3β/β-catenin signal regulation axis during the osteogenic differentiation of PDLSCs (Fig. 10).

## Discussion

As oral tissue-derived mesenchymal stem cells, PDLSCs are important candidate seed cells for bone tissue engineering to realize the regeneration of alveolar bone. Understanding the gene regulation mechanism of osteogenic differentiation in PDLSCs is critical for PDLSC-based bone regeneration. Microarray analysis based on a high-throughput platform has become a promising and efficient tool to search for meaningful genes that regulate the characteristics and functions of stem cells, and has been widely used by many scholars [32, 33]. The present study compared the gene expression profiles of undifferentiated and osteodifferentiated PDLSCs through a microarray assay, and then filtered 499 genes whose expression levels were altered significantly after osteogenic differentiation. It is possible that these differentially expressed genes participate in the regulation of osteogenic differentiation of PDLSCs. Indeed, *PSAT1*, one of these differentially expressed genes after osteogenic induction in PDLSCs, was proven to affect the osteogenic differentiation of PDLSCs through subsequent validation experiments. Thus, the results of the microarray assay provided clues for seeking potential regulators of osteogenic differentiation, and whether the other differentially expressed genes can regulate the osteogenesis of PDLSCs requires further study.

The present study proved that overexpressing *PSAT1* promoted the osteogenic differentiation of PDLSCs in vitro and in vivo, while knocking down *PSAT1* led to impaired osteogenesis. Therefore, we speculate that *PSAT1* is a positive regulator of the osteogenic differentiation of PDLSCs. In fact, there have been few studies on the regulation of *PSAT1* on the osteogenic differentiation of stem cells to date. Several scholars noted that *PSAT1* expression showed a sustained and significant change during the process of mineralization in osteoblasts and periodontal ligament cells, but the possible regulatory roles of *PSAT1* were not studied [29, 34]. Here, we comprehensively verified the regulatory effect of *PSAT1* on the osteogenic differentiation of PDLSCs first, providing new ideas for enhancing PDLSC-mediated bone regeneration. Notably, a few new clues suggest that *PSAT1* may affect other behaviour of stem cells: the ability of mouse ESCs to differentiate to ectodermal lineage was impaired when *Psat1* was knocked down [28]; the serine metabolism that *PSAT1* participated in controlled dental pulp stem cell ageing [30]. Therefore, *PSAT1* could be a potential important regulator of the biological characteristics of stem cells in many aspects, which is worthy of further research in the future.

The Akt signalling pathway has been reported to regulate the biological behaviour of stem cells [35, 36]. Generally, the activated Akt signal (phosphorylated Akt) inhibits GSK3β by phosphorylating it at Ser9, in turn promoting the stabilization and nuclear translocation of β-catenin, which could regulate the expression of downstream genes together with LEF/TCF and promote the osteogenic differentiation of stem cells [37, 38]. The present study proved that overexpressing *PSAT1* increased the phosphorylation level of Akt and promoted the activation of Akt/GSK3β/β-catenin signalling in PDLSCs, while knocking down *PSAT1* inhibited it. In addition, the inhibitor or activator of Akt signalling, LY294002 or SC79, reversed the effects of *PSAT1* overexpression or knockdown on the osteogenesis of PDLSCs, respectively. These results indicate that *PSAT1* could regulate the osteogenic differentiation of PDLSCs through the Akt/GSK3β/β-catenin signalling pathway. Our findings were similar to some previous studies on cancer; for example, *PSAT1* was reported to regulate β-catenin/cyclin D1 signalling in breast cancer cells [39], *PSAT1* was proven to affect the GSK3β/Snail pathway in esophageal cancer cells [40], and *PSAT1* could influence the PI3K/AKT pathway in cervical cancer cells [41]. Thus, *PSAT1* seems to have a wide influence on regulatory networks in different types of tissue cells. Notably, whether *PSAT1* affects the osteogenic differentiation of PDLSCs through other mechanisms remains unclear. Given that *PSAT1* is involved in the biosynthesis of serine in cells, several studies have supported that *PSAT1* regulates the behaviour of cancer cells by regulating serine production [42, 43]. A study on mouse ESCs revealed that *Psat1* affected the timing of ESC differentiation by regulating the intracellular α-Ketoglutarate level instead of serine [28]. Regardless, the regulatory mechanism of *PSAT1* may be diverse and needs further study.

The proliferation and self-renewal abilities of stem cells are also quite important for tissue engineering, because regeneration therapy generally requires a sufficient number of seed cells [44, 45]. Our results of CCK-8, EdU, and cell cycle assays proved that *PSAT1* positively regulated PDLSC proliferation, which could contribute to PDLSC-based bone tissue engineering. In fact, several scholars have found a connection between *PSAT1* and cell proliferation in some tumour cells. Nadia reported that the overexpression of *PSAT1* significantly stimulated the cell growth of colon cancer cells [24]. Yang proved that *PSAT1* strongly promoted the cell cycle progression and cell proliferation of non-small cell lung cancer cells [25]. Yan proved that downregulating *PSAT1* expression significantly inhibited the growth of esophageal cancer cells [46]. Notably, our results showed that the effect of *PSAT1* on PDLSCs proliferative activity was not as strong as previously reported in tumour cells [24, 25, 46]. This suggests that the regulation of *PSAT1* on cell behaviour could vary with cell type.

The present study found that *PSAT1* expression in PDLSCs decreased during osteogenic differentiation. Similarly, Hwang reported that *Psat1* was highly expressed in mouse ESCs and decreased during differentiation and suggested that maintaining *Psat1* levels could be essential for the self-renewal and pluripotency of mouse ESCs [28]. Therefore, we speculated that *PSAT1* might also be related to the stemness of PDLSCs, which may explain the decreased *PSAT1* expression in osteodifferentiated PDLSCs. In addition, we further proved that ATF4 could bind to the promoter region of *PSAT1* and regulate its translation in PDLSCs. As a transcription factor, ATF4 has been proven to be involved in a variety of physiological and metabolic processes and can regulate the biological behaviour of stem cells [47, 48]. Several studies revealed that *ATF4* could regulate the osteogenic differentiation of PDLSCs [49, 50]. Since the expression of *ATF4* in PDLSCs also decreased with osteogenic differentiation, we speculated that *ATF4* was one of the direct regulators that mediated the downregulation of *PSAT1* expression after osteogenic induction in PDLSCs. Our findings are similar to previous studies that reported that *ATF4* could influence *PSAT1* expression in some cancer cells [43, 51]. Considering that PDLSCs are quite different from these cancer cells, more exploration is required to explain the regulatory network involved in *PSAT1* in PDLSCs.

## Conclusion

Exploring the gene regulation mechanism for the osteogenic differentiation of PDLSCs is of great significance for PDLSC-based bone regeneration. The present study proved that *PSAT1* expression was altered significantly after osteogenic induction and could positively regulate the proliferation and osteogenic differentiation of PDLSCs in vitro and promoted PDLSC-based bone regeneration in vivo. The regulatory effects of *PSAT1* on osteogenesis could occur through the Akt/GSK3β/β-catenin signalling pathway. In addition, the transcription factor ATF4 could bind to the promotor region of *PSAT1* and regulate its expression. Collectively, *PSAT1* could be a potential important regulator of the osteogenic differentiation of PDLSCs and a candidate regulatory target for promoting PDLSC-based bone tissue engineering.

## Supplementary Information

**Additional file 1: Table S1.** The sequences of siRNAs.

**Additional file 2: Table S2.** The sequences of primers utilized for qRT-PCR.

**Additional file 3: Table S3.** The differentially expressed mRNAs in PDLSCs after osteogenic induction.

**Additional file 4: Figure S1.** Validation of microarray results in PDLSCs by qRT-PCR.

**Additional file 5: Figure S2.** The interference efficiency of using siRNA to interfere with target genes in PDLSCs.

**Additional file 6: Table S3.** Differentially expressed genes in PDLSCs that have co-expression relationships with PSAT1.

## Data Availability

All datasets generated for this study are included in the article.
