# Supplementary material for: PSAT1 positively regulates the osteogenic lineage differentiation of periodontal ligament stem cells through the ATF4/PSAT1/Akt/GSK3β/β-catenin axis
Source: J Transl Med. 2023 Feb 2;21:70. doi: 10.1186/s12967-022-03775-z (PMC9893676; doi:10.1186/s12967-022-03775-z)
Supplement: Supplementary file 1 — Additional file 1: Table S1. The sequences of siRNAs. [file 12967_2022_3775_MOESM1_ESM.docx]

**Table S1 The sequences of siRNAs**

| Target Gene | forward primer（5’-3’） | reverse primer（5’-3’） |
| --- | --- | --- |
| ***PIP*** | GCAAATTGCAGCCGTCGTT | TTCTCCGAACGTGTCACGT |
| ***FKBP5*** | GGAACAGACAGUCAAGCAAUG | UUGCUUGACUGUCUGUUCCUU |
| ***CORIN*** | GCAGUGUAAUGGCUACAAUTT | AUUGUAGCCAUUACACUGCTT |
| ***APOD*** | CGGAAAGAUCAAAGUGUUAAA | UAACACUUUGAUCUUUCCGUU |
| ***VLDLR*** | GGAUCUUCAGAAUUCUAAAGG | UUUAGAAUUCUGAAGAUCCUG |
| ***ASNS*** | GCUGUAAUGUGAAAGCAAAUA | UUUGCUUUCACAUUACAGCAU |
| ***SLC7A5*** | GGGUGAUGUGUCCAAUCUAGA | UAGAUUGGACACAUCACCCUU |
| ***PSAT1*** | CCAAGAAGUUUGGGACUAUTT | AUAGUCCCAAACUUCUUGGTT |
| ***ATF4*** | GGUGAACCCAAUUGGCCAUTT | AUGGCCAAUUGGGUUCACCTT |
| negative control | UUCUCCGAACGUGUCACGUTT | ACGUGACACGUUCGGAGAATT |
