# Supplementary material for: PSAT1 positively regulates the osteogenic lineage differentiation of periodontal ligament stem cells through the ATF4/PSAT1/Akt/GSK3β/β-catenin axis
Source: J Transl Med. 2023 Feb 2;21:70. doi: 10.1186/s12967-022-03775-z (PMC9893676; doi:10.1186/s12967-022-03775-z)
Supplement: Supplementary file 2 — Additional file 2: Table S2. The sequences of primers utilized for qRT-PCR. [file 12967_2022_3775_MOESM2_ESM.docx]

**Table S2 The sequences of primers utilized for qRT-PCR**

| **Gene** | **forward primer（5’-3’）** | **reverse primer（5’-3’）** |
| --- | --- | --- |
| ***GAPDH*** | GCACCGTCAAGGCTGAGAAC | TGGTGAAGACGCCAGTGGA |
| ***PIP*** | TACTGCCTGCCTATGTGACG | TCAGGGCAGATGCCTAATTC |
| ***PSAT1*** | ATTGGCTTGAAAGCAGGAAG | TCCCAAGTTTAGGGTGAACG |
| ***FKBP5*** | AGGGAGGCAAATACATGCAG | AAGGCAGCAAGGAGAAATGA |
| ***STC1*** | GGCGACCACCAAAGTCAAAC | GCAGTGACGCTCATAAGGGA |
| ***TNFSF18*** | AAGTGGCTCCCAATGCAAAC | GGTGTCCCCAACATGCAATTC |
| ***VCAM1*** | AATGCCTGGGAAGATGGTCG | GATGTGGTCCCCTCATTCGT |
| ***CORIN*** | CCTCCTCCGGTTCCTATTGC | CCAAAGGTTCACTCCCATTTGA |
| ***APOD*** | GAATCAAATCGAAGGTGAAGCCA | ACACGAGGGCATAGTTCTCAT |
| ***VLDLR*** | AGAAAAGCCAAATGTGAACCCT | CACTGCCGTCAACACAGTCT |
| ***ASNS*** | GGAAGACAGCCCCGATTTACT | AGCACGAACTGTTGTAATGTCA |
| ***SLC7A5*** | CCGTGAACTGCTACAGCGT | CTTCCCGATCTGGACGAAGC |
| ***ATF4*** | CTTGATGTCCCCCTTCGACC | CTTGTCGCTGGAGAACCCAT |
| ***COLI*** | GCTGATGATGCCAATGTGGTT | CCAGTCAGAGTGGCACATCTTG |
| ***ALP*** | TCCATCTGTAAAGGGCGGTAAT | AATACCAGCTACGCTGCATCAAG |
| ***RUNX2*** | GTTTCACCTTGACCATAACCGT | GGGACACCTACTCTCATACTGG |
