# Supplementary material for: PSAT1 positively regulates the osteogenic lineage differentiation of periodontal ligament stem cells through the ATF4/PSAT1/Akt/GSK3β/β-catenin axis
Source: J Transl Med. 2023 Feb 2;21:70. doi: 10.1186/s12967-022-03775-z (PMC9893676; doi:10.1186/s12967-022-03775-z)
Supplement: Supplementary file 3 — Additional file 3: Table S3. The differentially expressed mRNAs in PDLSCs after osteogenic induction. [file 12967_2022_3775_MOESM3_ESM.docx]

**Table S3 The differentially expressed mRNAs in PDLSCs after osteogenic induction**

| **Gene Symbol** | **Accession Number** | **Database Source** | **Fold Change** | ***p*-value** |
| --- | --- | --- | --- | --- |
| ***PIP*** | NM_002652 | RefSeq | 24.345794 | 0.000049 |
| ***FKBP5*** | NM_004117 | RefSeq | 16.359106 | 0.000049 |
| ***CORIN*** | NM_006587 | RefSeq | 8.917743 | 0.001161 |
| ***ADH1B*** | NM_000668 | RefSeq | 7.297252 | 0.005634 |
| ***APOD*** | NM_001647 | RefSeq | 6.332091 | 0.000061 |
| ***FRZB*** | NM_001463 | RefSeq | 6.326939 | 0.000049 |
| ***SAMHD1*** | NM_015474 | RefSeq | 5.355914 | 0.000049 |
| ***MAOA*** | NM_000240 | RefSeq | 5.111754 | 0.000183 |
| ***ABCA6*** | NM_080284 | RefSeq | 4.843544 | 0.000684 |
| ***ITGA10*** | NM_003637 | RefSeq | 4.659507 | 0.000318 |
| ***STC1*** | NM_003155 | RefSeq | 4.479721 | 0.001014 |
| ***AOX1*** | NM_001159 | RefSeq | 4.451833 | 0.000318 |
| ***TRNP1*** | NM_001013642 | RefSeq | 4.01628 | 0.001088 |
| ***METTL7A*** | NM_014033 | RefSeq | 3.830064 | 0.000049 |
| ***TIMP4*** | NM_003256 | RefSeq | 3.48772 | 0.008678 |
| ***GLUL*** | NM_001033044 | RefSeq | 3.28919 | 0.000477 |
| ***CD14*** | NM_001174104 | RefSeq | 3.283231 | 0.000782 |
| ***SPON1*** | NM_006108 | RefSeq | 3.034768 | 0.000953 |
| ***IRS2*** | NM_003749 | RefSeq | 2.964335 | 0.005145 |
| ***SPRY1*** | NM_005841 | RefSeq | 2.958302 | 0.000049 |
| ***AIM1*** | NM_001624 | RefSeq | 2.891143 | 0.000391 |
| ***MT1M*** | NM_176870 | RefSeq | 2.88806 | 0.000966 |
| ***NAV2*** | NM_001111018 | RefSeq | 2.838927 | 0.000171 |
| ***ANKRD1*** | NM_014391 | RefSeq | 2.823458 | 0.000452 |
| ***MOXD1*** | NM_015529 | RefSeq | 2.784916 | 0.002053 |
| ***CPM*** | NM_001005502 | RefSeq | 2.755793 | 0.003459 |
| ***HAS2*** | NM_005328 | RefSeq | 2.735396 | 0.004559 |
| ***GCNT1*** | NM_001097633 | RefSeq | 2.711554 | 0.000281 |
| ***GPM6B*** | NM_001001994 | RefSeq | 2.710407 | 0.000061 |
| ***ISM1*** | NM_080826 | RefSeq | 2.668733 | 0.0077 |
| ***MYPN*** | NM_001256267 | RefSeq | 2.648786 | 0.000171 |
| ***USP53*** | NM_019050 | RefSeq | 2.642993 | 0.009203 |
| ***IL12RB2*** | NM_001559 | RefSeq | 2.578947 | 0.014471 |
| ***INHBB*** | NM_002193 | RefSeq | 2.55361 | 0.001418 |
| ***IL1R1*** | NM_000877 | RefSeq | 2.493593 | 0.004082 |
| ***RAB27B*** | NM_004163 | RefSeq | 2.454843 | 0.002689 |
| ***COL11A1*** | NM_001190709 | RefSeq | 2.434632 | 0.004657 |
| ***DIO2*** | NM_000793 | RefSeq | 2.364571 | 0.045307 |
| ***ERCC6*** | NM_000124 | RefSeq | 2.345541 | 0.000049 |
| ***ANPEP*** | NM_001150 | RefSeq | 2.313802 | 0.000232 |
| ***ZBTB16*** | NM_001018011 | RefSeq | 2.278455 | 0.001222 |
| ***LEPR*** | NM_001003679 | RefSeq | 2.270161 | 0.003275 |
| ***RGS4*** | NM_001102445 | RefSeq | 2.266209 | 0.026338 |
| ***GPX3*** | NM_002084 | RefSeq | 2.247032 | 0.00121 |
| ***ANO3*** | NM_031418 | RefSeq | 2.231617 | 0.028758 |
| ***MT1E*** | NM_175617 | RefSeq | 2.228362 | 0.006392 |
| ***CFH*** | NM_000186 | RefSeq | 2.204213 | 0.000367 |
| ***SLC44A1*** | NM_080546 | RefSeq | 2.201651 | 0.00077 |
| ***RANBP3L*** | NM_001161429 | RefSeq | 2.18906 | 0.035211 |
| ***DUSP1*** | NM_004417 | RefSeq | 2.188223 | 0.000196 |
| ***PHKA1*** | NM_001122670 | RefSeq | 2.166058 | 0.003874 |
| ***TMEM150C*** | NM_001080506 | RefSeq | 2.164436 | 0.018333 |
| ***WISP1*** | NM_001204869 | RefSeq | 2.162866 | 0.00011 |
| ***DSP*** | NM_001008844 | RefSeq | 2.162119 | 0.00385 |
| ***ALCAM*** | NM_001243280 | RefSeq | 2.160589 | 0.000367 |
| ***ADAMTS2*** | NM_014244 | RefSeq | 2.140844 | 0.019738 |
| ***PGM2L1*** | NM_173582 | RefSeq | 2.084563 | 0.000196 |
| ***PTGER2*** | NM_000956 | RefSeq | 2.06699 | 0.005329 |
| ***SH3PXD2B*** | NM_001017995 | RefSeq | 2.062473 | 0.008299 |
| ***FMNL2*** | NM_052905 | RefSeq | 2.033533 | 0.004547 |
| ***PTGER4*** | NM_000958 | RefSeq | 2.021276 | 0.002567 |
| ***APOL2*** | NM_030882 | RefSeq | 2.019857 | 0.005622 |
| ***INSC*** | NM_001031853 | RefSeq | 2.018922 | 0.000306 |
| ***STARD4*** | NM_139164 | RefSeq | 2.013449 | 0.016206 |
| ***PRUNE2*** | NM_015225 | RefSeq | 2.011499 | 0.004864 |
| ***DPT*** | NM_001937 | RefSeq | 1.992294 | 0.001919 |
| ***NEXN*** | NM_001172309 | RefSeq | 1.977661 | 0.000379 |
| ***VIT*** | NM_001177969 | RefSeq | 1.969742 | 0.015106 |
| ***PRR5L*** | NM_001160167 | RefSeq | 1.966895 | 0.000049 |
| ***TRPA1*** | NM_007332 | RefSeq | 1.949406 | 0.021303 |
| ***GADD45B*** | NM_015675 | RefSeq | 1.941256 | 0.005207 |
| ***SNED1*** | NM_001080437 | RefSeq | 1.939172 | 0.010596 |
| ***BMP6*** | NM_001718 | RefSeq | 1.935814 | 0.002273 |
| ***LDLR*** | NM_000527 | RefSeq | 1.934627 | 0.006001 |
| ***ADH1C*** | NM_000669 | RefSeq | 1.929811 | 0.002823 |
| ***KREMEN1*** | NM_001039570 | RefSeq | 1.907249 | 0.000208 |
| ***IFI44L*** | NM_006820 | RefSeq | 1.907176 | 0.009264 |
| ***PCDH9*** | NM_020403 | RefSeq | 1.902585 | 0.005194 |
| ***NT5DC3*** | NM_001031701 | RefSeq | 1.894526 | 0.004889 |
| ***DIAPH2*** | NM_006729 | RefSeq | 1.892124 | 0.000477 |
| ***ADARB1*** | NM_015833 | RefSeq | 1.889972 | 0.00682 |
| ***IGFBP2*** | NM_000597 | RefSeq | 1.874487 | 0.001491 |
| ***LRRC8B*** | NM_001134476 | RefSeq | 1.872183 | 0.007407 |
| ***TMTC1*** | NM_001193451 | RefSeq | 1.870778 | 0.04527 |
| ***RGS17*** | NM_012419 | RefSeq | 1.869322 | 0.018492 |
| ***KCNIP3*** | NM_001034914 | RefSeq | 1.8662 | 0.002738 |
| ***FAM107A*** | NM_001076778 | RefSeq | 1.847837 | 0.001344 |
| ***ABCA8*** | NM_007168 | RefSeq | 1.843759 | 0.011745 |
| ***MKI67*** | NM_001145966 | RefSeq | 1.841423 | 0.000684 |
| ***SORT1*** | NM_001205228 | RefSeq | 1.838981 | 0.000098 |
| ***IRAK3*** | NM_001142523 | RefSeq | 1.829655 | 0.004082 |
| ***DNAJB4*** | NM_007034 | RefSeq | 1.828278 | 0.006099 |
| ***HIPK2*** | NM_001113239 | RefSeq | 1.82211 | 0.002835 |
| ***DUSP5*** | NM_004419 | RefSeq | 1.821688 | 0.004168 |
| ***MT1G*** | NM_005950 | RefSeq | 1.818809 | 0.019408 |
| ***TNFAIP3*** | NM_006290 | RefSeq | 1.801552 | 0.010682 |
| ***STXBP4*** | NM_178509 | RefSeq | 1.798784 | 0.001931 |
| ***CPPED1*** | NM_001099455 | RefSeq | 1.793682 | 0.005255 |
| ***SH3D19*** | NM_001009555 | RefSeq | 1.78634 | 0.000367 |
| ***RORB*** | NM_006914 | RefSeq | 1.781145 | 0.043192 |
| ***SHC3*** | NM_016848 | RefSeq | 1.779299 | 0.005585 |
| ***SSH2*** | NM_033389 | RefSeq | 1.777331 | 0.00022 |
| ***PTK2B*** | NM_004103 | RefSeq | 1.77705 | 0.006771 |
| ***ABCA13*** | NM_152701 | RefSeq | 1.77455 | 0.004033 |
| ***HIP1*** | NM_001243198 | RefSeq | 1.771143 | 0.005329 |
| ***PROS1*** | NM_000313 | RefSeq | 1.766081 | 0.029443 |
| ***MT1X*** | NM_005952 | RefSeq | 1.75986 | 0.048814 |
| ***SP110*** | NM_001185015 | RefSeq | 1.752622 | 0.005463 |
| ***EPHA4*** | NM_004438 | RefSeq | 1.751506 | 0.018357 |
| ***PDE7B*** | NM_018945 | RefSeq | 1.748165 | 0.000721 |
| ***C5AR2*** | NM_018485 | RefSeq | 1.747612 | 0.00264 |
| ***MMP19*** | NM_002429 | RefSeq | 1.746922 | 0.005512 |
| ***DTNA*** | NM_032975 | RefSeq | 1.73991 | 0.003324 |
| ***ARRDC2*** | NM_001025604 | RefSeq | 1.736669 | 0.009765 |
| ***RDH10*** | NM_172037 | RefSeq | 1.736555 | 0.007407 |
| ***ANKH*** | NM_054027 | RefSeq | 1.735036 | 0.039012 |
| ***EPS8*** | NM_004447 | RefSeq | 1.734311 | 0.00143 |
| ***ARHGAP18*** | NM_033515 | RefSeq | 1.730044 | 0.027744 |
| ***NEGR1*** | NM_173808 | RefSeq | 1.728558 | 0.001344 |
| ***TGFBR2*** | NM_001024847 | RefSeq | 1.724071 | 0.000391 |
| ***SEC14L2*** | NM_001204204 | RefSeq | 1.71816 | 0.000171 |
| ***TSPAN5*** | NM_005723 | RefSeq | 1.71439 | 0.000159 |
| ***COL3A1*** | NM_000090 | RefSeq | 1.7125 | 0.02833 |
| ***ZFP36*** | NM_003407 | RefSeq | 1.711612 | 0.000122 |
| ***NRP2*** | NM_201266 | RefSeq | 1.710526 | 0.000147 |
| ***FBXO32*** | NM_001242463 | RefSeq | 1.699033 | 0.00077 |
| ***PTGFR*** | NM_000959 | RefSeq | 1.692704 | 0.005023 |
| ***NOTCH2*** | NM_001200001 | RefSeq | 1.678115 | 0.00495 |
| ***C18orf54*** | NM_173529 | RefSeq | 1.669515 | 0.002811 |
| ***ABHD2*** | NM_007011 | RefSeq | 1.66241 | 0.004033 |
| ***CRISPLD2*** | NM_031476 | RefSeq | 1.657159 | 0.048326 |
| ***ADH1A*** | NM_000667 | RefSeq | 1.655725 | 0.023527 |
| ***TANC2*** | NM_025185 | RefSeq | 1.654876 | 0.005732 |
| ***MLKL*** | NM_001142497 | RefSeq | 1.654286 | 0.001797 |
| ***INMT*** | NM_006774 | RefSeq | 1.650198 | 0.005414 |
| ***COL10A1*** | NM_000493 | RefSeq | 1.649385 | 0.000208 |
| ***GPSM2*** | NM_013296 | RefSeq | 1.646725 | 0.004987 |
| ***FMO3*** | NM_001002294 | RefSeq | 1.646304 | 0.000171 |
| ***PRKD1*** | NM_002742 | RefSeq | 1.643209 | 0.000318 |
| ***ZFP36L2*** | NM_006887 | RefSeq | 1.638387 | 0.000122 |
| ***PLXNA2*** | NM_025179 | RefSeq | 1.635999 | 0.001577 |
| ***PIK3R1*** | NM_001242466 | RefSeq | 1.634798 | 0.000966 |
| ***ANLN*** | NM_018685 | RefSeq | 1.634771 | 0.035896 |
| ***NFXL1*** | NM_152995 | RefSeq | 1.634024 | 0.007895 |
| ***PRICKLE2*** | NM_198859 | RefSeq | 1.633974 | 0.000391 |
| ***ANGPTL4*** | NM_139314 | RefSeq | 1.632727 | 0.013236 |
| ***FBLN1*** | NM_001996 | RefSeq | 1.629799 | 0.01727 |
| ***RNF157*** | NM_052916 | RefSeq | 1.6284 | 0.021755 |
| ***NR4A3*** | NM_173199 | RefSeq | 1.62792 | 0.006001 |
| ***PER1*** | NM_002616 | RefSeq | 1.624689 | 0.000061 |
| ***CMKLR1*** | ENST00000397688 | ENSEMBL | 1.622952 | 0.038206 |
| ***MSMO1*** | NM_001017369 | RefSeq | 1.618993 | 0.026069 |
| ***COX11*** | NM_001162861 | RefSeq | 1.61877 | 0.02057 |
| ***ZHX3*** | NM_015035 | RefSeq | 1.613788 | 0.000208 |
| ***MT1B*** | NM_005947 | RefSeq | 1.610916 | 0.010511 |
| ***TPX2*** | NM_012112 | RefSeq | 1.609412 | 0.0033 |
| ***CLSPN*** | NM_001190481 | RefSeq | 1.609158 | 0.041958 |
| ***NFIA*** | NM_001134673 | RefSeq | 1.60692 | 0.004412 |
| ***ALPL*** | NM_000478 | RefSeq | 1.603031 | 0.020386 |
| ***PUS7L*** | NM_001098614 | RefSeq | 1.602795 | 0.003801 |
| ***PALLD*** | NM_016081 | RefSeq | 1.602421 | 0.031337 |
| ***SPTLC3*** | NM_018327 | RefSeq | 1.600969 | 0.000794 |
| ***SMIM10*** | NM_001163438 | RefSeq | 1.600784 | 0.01496 |
| ***GPR89A*** | NM_001097612 | RefSeq | 1.593177 | 0.009521 |
| ***STXBP5*** | NM_001127715 | RefSeq | 1.592968 | 0.007345 |
| ***INS*** | NM_000207 | RefSeq | 1.591275 | 0.008836 |
| ***ANGPTL5*** | NM_178127 | RefSeq | 1.590532 | 0.000477 |
| ***OPN3*** | NM_014322 | RefSeq | 1.589322 | 0.000477 |
| ***CENPI*** | NM_006733 | RefSeq | 1.588203 | 0.008372 |
| ***MAP3K5*** | NM_005923 | RefSeq | 1.586431 | 0.002909 |
| ***MYO1E*** | NM_004998 | RefSeq | 1.585917 | 0.010193 |
| ***CTSK*** | NM_000396 | RefSeq | 1.584002 | 0.011085 |
| ***B3GNT5*** | NM_032047 | RefSeq | 1.581704 | 0.000183 |
| ***NFIL3*** | NM_005384 | RefSeq | 1.580899 | 0.004608 |
| ***LIX1L*** | NM_153713 | RefSeq | 1.578952 | 0.012906 |
| ***WSCD1*** | NM_015253 | RefSeq | 1.578139 | 0.012369 |
| ***TMEM100*** | NM_001099640 | RefSeq | 1.577185 | 0.029748 |
| ***RPS6KA2*** | NM_001006932 | RefSeq | 1.57518 | 0.000831 |
| ***COPS8*** | NM_006710 | RefSeq | 1.57455 | 0.002432 |
| ***FBN2*** | NM_001999 | RefSeq | 1.572301 | 0.012149 |
| ***APOB*** | NM_000384 | RefSeq | 1.572166 | 0.009863 |
| ***COL8A1*** | NM_001850 | RefSeq | 1.569797 | 0.01089 |
| ***HMGA2*** | NM_003484 | RefSeq | 1.568909 | 0.006233 |
| ***CHEK1*** | NM_001114121 | RefSeq | 1.566981 | 0.001088 |
| ***AKAP13*** | NM_144767 | RefSeq | 1.557105 | 0.001112 |
| ***ABCA9*** | NM_080283 | RefSeq | 1.556425 | 0.010071 |
| ***AUTS2*** | NM_001127231 | RefSeq | 1.553749 | 0.045453 |
| ***SERPING1*** | NM_000062 | RefSeq | 1.549338 | 0.001858 |
| ***COMP*** | NM_000095 | RefSeq | 1.548884 | 0.005414 |
| ***DIAPH1*** | NM_001079812 | RefSeq | 1.545311 | 0.004168 |
| ***KLF9*** | NM_001206 | RefSeq | 1.544062 | 0.002151 |
| ***ZNF106*** | NM_022473 | RefSeq | 1.541342 | 0.033341 |
| ***ELK3*** | NM_005230 | RefSeq | 1.540017 | 0.000354 |
| ***PHF15*** | NM_015288 | RefSeq | 1.537017 | 0.000171 |
| ***USP13*** | NM_003940 | RefSeq | 1.535436 | 0.031924 |
| ***GPR89C*** | NM_001097616 | RefSeq | 1.53445 | 0.010169 |
| ***NCOA3*** | NM_006534 | RefSeq | 1.533445 | 0.007248 |
| ***ITGA5*** | NM_002205 | RefSeq | 1.530896 | 0.002897 |
| ***PMP22*** | NM_000304 | RefSeq | 1.530798 | 0.003911 |
| ***SLC1A1*** | NM_004170 | RefSeq | 1.530621 | 0.01265 |
| ***FLVCR2*** | NM_001195283 | RefSeq | 1.530292 | 0.000342 |
| ***ANKRD35*** | NM_144698 | RefSeq | 1.530035 | 0.002224 |
| ***ESCO2*** | NM_001017420 | RefSeq | 1.526187 | 0.007455 |
| ***KIF11*** | NM_004523 | RefSeq | 1.524049 | 0.022953 |
| ***OLFML2A*** | NM_182487 | RefSeq | 1.523567 | 0.004033 |
| ***PTPLAD2*** | NM_001010915 | RefSeq | 1.517116 | 0.012772 |
| ***LIPH*** | NM_139248 | RefSeq | 1.516611 | 0.014642 |
| ***RASD1*** | NM_001199989 | RefSeq | 1.515842 | 0.001406 |
| ***FSTL3*** | NM_005860 | RefSeq | 1.514716 | 0.009105 |
| ***FRAS1*** | NM_001166133 | RefSeq | 1.513855 | 0.019836 |
| ***FOXC2*** | NM_005251 | RefSeq | 1.512844 | 0.005427 |
| ***SQRDL*** | NM_021199 | RefSeq | 1.511838 | 0.028269 |
| ***SAA1*** | NM_000331 | RefSeq | 1.508155 | 0.009423 |
| ***LPCAT3*** | NM_005768 | RefSeq | 1.50741 | 0.003434 |
| ***LTBP1*** | NM_000627 | RefSeq | 1.506375 | 0.013273 |
| ***EMP2*** | NM_001424 | RefSeq | 1.504986 | 0.007626 |
| ***FOXO3*** | NM_001455 | RefSeq | 1.503409 | 0.0165 |
| ***GTF2A1L*** | NM_001193487 | RefSeq | 1.501481 | 0.000746 |
| ***ENTPD1*** | NM_001164182 | RefSeq | 1.501167 | 0.004534 |
| ***CNTNAP1*** | NM_003632 | RefSeq | -1.500657 | 0.002298 |
| ***PTER*** | NM_001001484 | RefSeq | -1.501689 | 0.001161 |
| ***UGGT2*** | NM_020121 | RefSeq | -1.50602 | 0.003544 |
| ***PELI2*** | NM_021255 | RefSeq | -1.506155 | 0.003495 |
| ***TLK1*** | NM_012290 | RefSeq | -1.507119 | 0.001247 |
| ***AIMP2*** | NM_006303 | RefSeq | -1.508691 | 0.032119 |
| ***PTK7*** | NM_002821 | RefSeq | -1.509253 | 0.005805 |
| ***ZNF419*** | NM_001098491 | RefSeq | -1.509367 | 0.002713 |
| ***HMOX1*** | NM_002133 | RefSeq | -1.512284 | 0.021547 |
| ***LPCAT2*** | NM_017839 | RefSeq | -1.51533 | 0.007932 |
| ***UHRF1BP1*** | NM_017754 | RefSeq | -1.51574 | 0.000501 |
| ***LARP6*** | NM_018357 | RefSeq | -1.516554 | 0.000122 |
| ***E2F5*** | NM_001083588 | RefSeq | -1.51757 | 0.003691 |
| ***UNC5B*** | NM_001244889 | RefSeq | -1.518668 | 0.038365 |
| ***C16orf45*** | NM_033201 | RefSeq | -1.521935 | 0.000672 |
| ***NR3C1*** | NM_001018077 | RefSeq | -1.524334 | 0.000134 |
| ***LAYN*** | NM_178834 | RefSeq | -1.528231 | 0.036299 |
| ***RNF41*** | NM_001242826 | RefSeq | -1.528839 | 0.002176 |
| ***PSPH*** | NM_004577 | RefSeq | -1.531294 | 0.00495 |
| ***E2F7*** | NM_203394 | RefSeq | -1.531554 | 0.009851 |
| ***GALM*** | NM_138801 | RefSeq | -1.532704 | 0.000147 |
| ***TRPC4*** | NM_001135955 | RefSeq | -1.533643 | 0.004045 |
| ***SLC31A1*** | NM_001859 | RefSeq | -1.533883 | 0.00077 |
| ***UCHL1*** | NM_004181 | RefSeq | -1.534058 | 0.000721 |
| ***NGRN*** | NM_001033088 | RefSeq | -1.536436 | 0.014703 |
| ***MKNK2*** | NM_017572 | RefSeq | -1.536439 | 0.004779 |
| ***BCL2*** | NM_000633 | RefSeq | -1.538376 | 0.048044 |
| ***WNT5A*** | NM_001256105 | RefSeq | -1.538771 | 0.047226 |
| ***EIF5*** | NM_001969 | RefSeq | -1.54039 | 0.000196 |
| ***LEPRE1*** | NM_001146289 | RefSeq | -1.541954 | 0.00088 |
| ***VASN*** | NM_138440 | RefSeq | -1.543522 | 0.000868 |
| ***DHRS3*** | NM_004753 | RefSeq | -1.546479 | 0.000134 |
| ***LARS*** | NM_020117 | RefSeq | -1.548164 | 0.019494 |
| ***FOPNL*** | NM_144600 | RefSeq | -1.548505 | 0.040235 |
| ***PHF17*** | NM_199320 | RefSeq | -1.551169 | 0.027732 |
| ***SLFN5*** | NM_144975 | RefSeq | -1.554994 | 0.002102 |
| ***LHX8*** | NM_001001933 | RefSeq | -1.556672 | 0.002334 |
| ***NINJ1*** | NM_004148 | RefSeq | -1.55882 | 0.004815 |
| ***FAM155A*** | NM_001080396 | RefSeq | -1.560159 | 0.018052 |
| ***EPHB2*** | NM_004442 | RefSeq | -1.564809 | 0.009215 |
| ***PGD*** | NM_002631 | RefSeq | -1.564954 | 0.018088 |
| ***MFAP4*** | NM_001198695 | RefSeq | -1.565202 | 0.010328 |
| ***CES2*** | NM_003869 | RefSeq | -1.565585 | 0.00077 |
| ***NDNF*** | NM_024574 | RefSeq | -1.5663 | 0.001088 |
| ***IFI30*** | NM_006332 | RefSeq | -1.570828 | 0.045221 |
| ***BDKRB2*** | NM_000623 | RefSeq | -1.572642 | 0.001088 |
| ***MAB21L1*** | NM_005584 | RefSeq | -1.572721 | 0.00088 |
| ***FAM46A*** | NM_017633 | RefSeq | -1.577787 | 0.000293 |
| ***TLR3*** | NM_003265 | RefSeq | -1.582166 | 0.028844 |
| ***LDB2*** | NM_001130834 | RefSeq | -1.582779 | 0.000501 |
| ***PDCD1LG2*** | NM_025239 | RefSeq | -1.585634 | 0.014434 |
| ***EIF1B*** | NM_005875 | RefSeq | -1.588106 | 0.01727 |
| ***CTSC*** | NM_001114173 | RefSeq | -1.588479 | 0.038047 |
| ***HSPA5*** | NM_005347 | RefSeq | -1.590328 | 0.005867 |
| ***NOVA1*** | NM_002515 | RefSeq | -1.591155 | 0.036263 |
| ***NEDD4L*** | NM_001144968 | RefSeq | -1.596927 | 0.014996 |
| ***CACNA2D3*** | NM_018398 | RefSeq | -1.597116 | 0.007822 |
| ***ADRA1D*** | NM_000678 | RefSeq | -1.597523 | 0.003471 |
| ***GRIA3*** | NM_000828 | RefSeq | -1.597942 | 0.00363 |
| ***TP53*** | NM_000546 | RefSeq | -1.598425 | 0.005378 |
| ***P4HA2*** | NM_001017973 | RefSeq | -1.600035 | 0.001283 |
| ***ARHGAP42*** | NM_152432 | RefSeq | -1.601606 | 0.002053 |
| ***ELFN1*** | NM_001128636 | RefSeq | -1.602386 | 0.001748 |
| ***PYROXD1*** | NM_024854 | RefSeq | -1.602675 | 0.00539 |
| ***CD44*** | NM_001001391 | RefSeq | -1.603484 | 0.002762 |
| ***C5orf28*** | NM_022483 | RefSeq | -1.605275 | 0.010218 |
| ***AKR1C1*** | NM_001353 | RefSeq | -1.60546 | 0.021071 |
| ***LONP1*** | NM_004793 | RefSeq | -1.606512 | 0.000171 |
| ***C15orf65*** | NM_001198784 | RefSeq | -1.607721 | 0.00253 |
| ***BTG2*** | NM_006763 | RefSeq | -1.609756 | 0.00407 |
| ***TALDO1*** | NM_006755 | RefSeq | -1.61206 | 0.014642 |
| ***FOXD1*** | NM_004472 | RefSeq | -1.615419 | 0.018027 |
| ***XBP1*** | NM_001079539 | RefSeq | -1.623546 | 0.00759 |
| ***EDA2R*** | NM_001199687 | RefSeq | -1.62421 | 0.008335 |
| ***FAT1*** | NM_005245 | RefSeq | -1.625535 | 0.009032 |
| ***DDIT4*** | NM_019058 | RefSeq | -1.631355 | 0.00209 |
| ***RPS5*** | NM_001009 | RefSeq | -1.632773 | 0.040772 |
| ***CALB2*** | NM_001740 | RefSeq | -1.634494 | 0.028245 |
| ***ALDH2*** | NM_000690 | RefSeq | -1.637016 | 0.015094 |
| ***RALGPS2*** | NM_152663 | RefSeq | -1.637107 | 0.001919 |
| ***IFRD1*** | NM_001007245 | RefSeq | -1.641469 | 0.002273 |
| ***ME1*** | NM_002395 | RefSeq | -1.644486 | 0.001638 |
| ***IL20RB*** | NM_144717 | RefSeq | -1.64783 | 0.01903 |
| ***SCN9A*** | NM_002977 | RefSeq | -1.648977 | 0.000293 |
| ***HIST1H4H*** | NM_003543 | RefSeq | -1.660328 | 0.015229 |
| ***AGA*** | NM_000027 | RefSeq | -1.664397 | 0.009802 |
| ***COX8A*** | NM_004074 | RefSeq | -1.66457 | 0.010914 |
| ***KCTD15*** | NM_001129994 | RefSeq | -1.6675 | 0.005512 |
| ***EPAS1*** | NM_001430 | RefSeq | -1.67546 | 0.026424 |
| ***C10orf107*** | NM_173554 | RefSeq | -1.679948 | 0.001222 |
| ***GTPBP2*** | NM_019096 | RefSeq | -1.680786 | 0.000856 |
| ***SYT11*** | NM_152280 | RefSeq | -1.683757 | 0.001161 |
| ***EXT1*** | NM_000127 | RefSeq | -1.690711 | 0.000171 |
| ***HYOU1*** | NM_001130991 | RefSeq | -1.690895 | 0.000599 |
| ***PTHLH*** | NM_002820 | RefSeq | -1.695382 | 0.012919 |
| ***ENPP2*** | NM_001040092 | RefSeq | -1.695514 | 0.010609 |
| ***NCKAP5*** | NM_207363 | RefSeq | -1.698452 | 0.002909 |
| ***SLC14A1*** | NM_015865 | RefSeq | -1.701273 | 0.04109 |
| ***OSR2*** | NM_001142462 | RefSeq | -1.703245 | 0.017502 |
| ***MKX*** | NM_001242702 | RefSeq | -1.704521 | 0.012711 |
| ***TIGD2*** | NM_145715 | RefSeq | -1.704762 | 0.003691 |
| ***LRP12*** | NM_001135703 | RefSeq | -1.704913 | 0.000147 |
| ***TIMP3*** | NM_000362 | RefSeq | -1.707403 | 0.003544 |
| ***BDKRB1*** | NM_000710 | RefSeq | -1.71079 | 0.001552 |
| ***CYFIP2*** | NM_001037332 | RefSeq | -1.717021 | 0.010071 |
| ***PAPPA2*** | NM_020318 | RefSeq | -1.7191 | 0.010902 |
| ***FLT1*** | NM_001160031 | RefSeq | -1.724556 | 0.007394 |
| ***SAMD5*** | NM_001030060 | RefSeq | -1.73165 | 0.002017 |
| ***EPRS*** | NM_004446 | RefSeq | -1.731948 | 0.004229 |
| ***ALPK2*** | NM_052947 | RefSeq | -1.732384 | 0.004119 |
| ***PRSS12*** | NM_003619 | RefSeq | -1.73663 | 0.00044 |
| ***TRIM16*** | NM_006470 | RefSeq | -1.741703 | 0.001833 |
| ***SLC22A15*** | NM_018420 | RefSeq | -1.743226 | 0.002579 |
| ***BCAT1*** | NM_001178091 | RefSeq | -1.744665 | 0.001247 |
| ***GPC6*** | NM_005708 | RefSeq | -1.751219 | 0.005292 |
| ***RSL24D1*** | NM_016304 | RefSeq | -1.752254 | 0.010071 |
| ***MSTN*** | NM_005259 | RefSeq | -1.752352 | 0.008005 |
| ***SERPINH1*** | NM_001207014 | RefSeq | -1.756985 | 0.00209 |
| ***FAM171B*** | NM_177454 | RefSeq | -1.757569 | 0.001564 |
| ***DRAM1*** | NM_018370 | RefSeq | -1.759611 | 0.003715 |
| ***TRIM16L*** | NM_001037330 | RefSeq | -1.761435 | 0.001577 |
| ***TNFRSF10B*** | NM_003842 | RefSeq | -1.77042 | 0.003691 |
| ***FAIM2*** | NM_012306 | RefSeq | -1.772336 | 0.003862 |
| ***PLAU*** | NM_001145031 | RefSeq | -1.772962 | 0.01914 |
| ***HSPA4L*** | NM_014278 | RefSeq | -1.774202 | 0.001283 |
| ***CTH*** | NM_001190463 | RefSeq | -1.779947 | 0.017844 |
| ***VEGFA*** | NM_001025366 | RefSeq | -1.783944 | 0.005145 |
| ***RAB32*** | NM_006834 | RefSeq | -1.784689 | 0.014287 |
| ***NR1D2*** | NM_001145425 | RefSeq | -1.786597 | 0.000061 |
| ***NUPR1*** | NM_001042483 | RefSeq | -1.790154 | 0.000367 |
| ***EXOSC6*** | NM_058219 | RefSeq | -1.801119 | 0.004363 |
| ***COLEC12*** | NM_130386 | RefSeq | -1.801288 | 0.005427 |
| ***EYA4*** | NM_004100 | RefSeq | -1.804522 | 0.012906 |
| ***LOXL2*** | NM_002318 | RefSeq | -1.805202 | 0.002359 |
| ***MEST*** | NM_001253900 | RefSeq | -1.814841 | 0.000464 |
| ***CPE*** | NM_001873 | RefSeq | -1.818454 | 0.000367 |
| ***RGMB*** | NM_001012761 | RefSeq | -1.822109 | 0.004987 |
| ***KCND3*** | NM_004980 | RefSeq | -1.825458 | 0.006184 |
| ***GALNT16*** | NM_001168368 | RefSeq | -1.827717 | 0.000721 |
| ***NGF*** | NM_002506 | RefSeq | -1.82957 | 0.000391 |
| ***XAF1*** | NM_017523 | RefSeq | -1.831173 | 0.002151 |
| ***PTGS2*** | NM_000963 | RefSeq | -1.831349 | 0.015241 |
| ***DCLK2*** | NM_001040260 | RefSeq | -1.834342 | 0.001491 |
| ***ADAMTS6*** | NM_197941 | RefSeq | -1.839629 | 0.004363 |
| ***SLC47A1*** | NM_018242 | RefSeq | -1.840723 | 0.040076 |
| ***EIF2S2*** | NM_003908 | RefSeq | -1.841848 | 0.00979 |
| ***ANKRD29*** | NM_173505 | RefSeq | -1.846158 | 0.001601 |
| ***AKAP6*** | NM_004274 | RefSeq | -1.847526 | 0.000232 |
| ***PMAIP1*** | NM_021127 | RefSeq | -1.85568 | 0.008934 |
| ***HERPUD1*** | NM_001010990 | RefSeq | -1.864166 | 0.000049 |
| ***PER3*** | NM_016831 | RefSeq | -1.87162 | 0.000709 |
| ***AJUBA*** | NM_032876 | RefSeq | -1.875605 | 0.001369 |
| ***GARS*** | NM_002047 | RefSeq | -1.876245 | 0.000464 |
| ***TNFAIP6*** | NM_007115 | RefSeq | -1.876902 | 0.003862 |
| ***SERPINE2*** | NM_001136528 | RefSeq | -1.878168 | 0.005207 |
| ***ZNF773*** | NM_198542 | RefSeq | -1.881457 | 0.00132 |
| ***TARSL2*** | NM_152334 | RefSeq | -1.881544 | 0.000367 |
| ***PLA2G4A*** | NM_024420 | RefSeq | -1.886731 | 0.000892 |
| ***GRPEL2*** | NM_152407 | RefSeq | -1.894375 | 0.004547 |
| ***PRNP*** | NM_000311 | RefSeq | -1.906058 | 0.000281 |
| ***DOK6*** | NM_152721 | RefSeq | -1.911135 | 0.046101 |
| ***MTHFD1L*** | NM_001242767 | RefSeq | -1.915375 | 0.001308 |
| ***PAPPA-AS1*** | ENST00000445861 | ENSEMBL | -1.917144 | 0.000892 |
| ***SLC7A1*** | NM_003045 | RefSeq | -1.918704 | 0.000171 |
| ***TNFRSF19*** | NM_018647 | RefSeq | -1.920721 | 0.005414 |
| ***CBS*** | NM_001178009 | RefSeq | -1.924266 | 0.000061 |
| ***P4HA1*** | NM_000917 | RefSeq | -1.928025 | 0.010071 |
| ***NRG1*** | NM_001160008 | RefSeq | -1.92909 | 0.001051 |
| ***MOCOS*** | NM_017947 | RefSeq | -1.941517 | 0.001491 |
| ***SOX4*** | NM_003107 | RefSeq | -1.942939 | 0.027475 |
| ***AVPI1*** | NM_021732 | RefSeq | -1.94849 | 0.006245 |
| ***GDF15*** | NM_004864 | RefSeq | -1.949621 | 0.000709 |
| ***ULBP1*** | NM_025218 | RefSeq | -1.950172 | 0.00594 |
| ***CEBPG*** | NM_001252296 | RefSeq | -1.956089 | 0.000318 |
| ***OGN*** | NM_014057 | RefSeq | -1.957247 | 0.005928 |
| ***TGFB2*** | NM_001135599 | RefSeq | -1.961266 | 0.014923 |
| ***EPGN*** | NM_001013442 | RefSeq | -1.964152 | 0.003642 |
| ***CARS*** | NM_001014437 | RefSeq | -1.964475 | 0.004742 |
| ***CSTA*** | NM_005213 | RefSeq | -1.969806 | 0.004657 |
| ***NARS*** | NM_004539 | RefSeq | -1.970855 | 0.002615 |
| ***SLC2A1*** | NM_006516 | RefSeq | -1.973866 | 0.001369 |
| ***BGN*** | NM_001711 | RefSeq | -1.979131 | 0.005329 |
| ***ATF4*** | NM_182810 | RefSeq | -1.979171 | 0.000868 |
| ***HSPA13*** | NM_006948 | RefSeq | -1.982295 | 0.005097 |
| ***LSAMP*** | NM_002338 | RefSeq | -1.997826 | 0.030922 |
| ***TRIB3*** | NM_021158 | RefSeq | -2.019908 | 0.000733 |
| ***PRKG1*** | NM_001098512 | RefSeq | -2.026849 | 0.026326 |
| ***STAC*** | NM_003149 | RefSeq | -2.026921 | 0.000587 |
| ***CDH2*** | NM_001792 | RefSeq | -2.039955 | 0.000709 |
| ***ARL4C*** | NM_005737 | RefSeq | -2.040911 | 0.003312 |
| ***ITIH5*** | NM_001001851 | RefSeq | -2.042605 | 0.018247 |
| ***ALDH1A3*** | NM_000693 | RefSeq | -2.048085 | 0.002298 |
| ***ZNF521*** | NM_015461 | RefSeq | -2.050156 | 0.01133 |
| ***ALDH3A2*** | NM_000382 | RefSeq | -2.055783 | 0.020423 |
| ***PAPPA*** | NM_002581 | RefSeq | -2.070063 | 0.033684 |
| ***TARS*** | NM_152295 | RefSeq | -2.079972 | 0.004143 |
| ***ADAM12*** | NM_003474 | RefSeq | -2.091924 | 0.001736 |
| ***ALDH1L2*** | NM_001034173 | RefSeq | -2.098537 | 0.000061 |
| ***SLC6A9*** | NM_001024845 | RefSeq | -2.104413 | 0.003691 |
| ***KLHL5*** | NM_001007075 | RefSeq | -2.115271 | 0.000049 |
| ***KRTAP1-1*** | ENST00000543328 | ENSEMBL | -2.121028 | 0.036678 |
| ***CAMK1D*** | NM_020397 | RefSeq | -2.127093 | 0.005928 |
| ***KCTD16*** | NM_020768 | RefSeq | -2.129943 | 0.006588 |
| ***PIEZO2*** | NM_022068 | RefSeq | -2.138051 | 0.000049 |
| ***SEMA3A*** | NM_006080 | RefSeq | -2.142476 | 0.000061 |
| ***PLK2*** | NM_001252226 | RefSeq | -2.149653 | 0.000587 |
| ***APOL6*** | NM_030641 | RefSeq | -2.151472 | 0.006001 |
| ***CXCL12*** | NM_000609 | RefSeq | -2.153885 | 0.011452 |
| ***HMCN1*** | NM_031935 | RefSeq | -2.157443 | 0.006099 |
| ***FER1L6*** | NM_001039112 | RefSeq | -2.158604 | 0.036287 |
| ***SULF2*** | NM_001161841 | RefSeq | -2.160593 | 0.003715 |
| ***LRRC15*** | NM_130830 | RefSeq | -2.169903 | 0.000538 |
| ***C1orf51*** | NM_144697 | RefSeq | -2.21748 | 0.009704 |
| ***RASSF2*** | NM_014737 | RefSeq | -2.229023 | 0.000208 |
| ***PCK2*** | NM_001018073 | RefSeq | -2.24046 | 0.000049 |
| ***CDH13*** | NM_001220488 | RefSeq | -2.268211 | 0.000318 |
| ***TUBE1*** | NM_016262 | RefSeq | -2.275107 | 0.000171 |
| ***SYTL2*** | NM_001162951 | RefSeq | -2.282749 | 0.00022 |
| ***ITGA11*** | NM_001004439 | RefSeq | -2.292198 | 0.041212 |
| ***SLC1A4*** | NM_001193493 | RefSeq | -2.305069 | 0.000147 |
| ***F2RL2*** | NM_004101 | RefSeq | -2.321028 | 0.000587 |
| ***HSPA9*** | NM_004134 | RefSeq | -2.324048 | 0.000892 |
| ***MSC*** | NM_005098 | RefSeq | -2.324089 | 0.000049 |
| ***TCEA1*** | NM_006756 | RefSeq | -2.33399 | 0.000086 |
| ***GUCY1A2*** | NM_000855 | RefSeq | -2.364006 | 0.008213 |
| ***SLC16A4*** | NM_001201546 | RefSeq | -2.366415 | 0.012075 |
| ***SARS*** | NM_006513 | RefSeq | -2.367471 | 0.000648 |
| ***ARHGEF2*** | NM_001162383 | RefSeq | -2.380054 | 0.000306 |
| ***IGFBP5*** | NM_000599 | RefSeq | -2.391837 | 0.014654 |
| ***UGCG*** | NM_003358 | RefSeq | -2.395826 | 0.000098 |
| ***PSG1*** | NM_006905 | RefSeq | -2.411931 | 0.03185 |
| ***SLIT2*** | NM_004787 | RefSeq | -2.433289 | 0.000171 |
| ***SHMT2*** | NM_001166356 | RefSeq | -2.434824 | 0.000147 |
| ***SH2D4A*** | NM_001174159 | RefSeq | -2.45398 | 0.000061 |
| ***KIT*** | NM_000222 | RefSeq | -2.45889 | 0.000147 |
| ***YARS*** | NM_003680 | RefSeq | -2.487585 | 0.000966 |
| ***FLRT2*** | NM_013231 | RefSeq | -2.489977 | 0.004021 |
| ***AARS*** | NM_001605 | RefSeq | -2.54259 | 0.000049 |
| ***IARS*** | NM_002161 | RefSeq | -2.578442 | 0.000171 |
| ***ANGPTL2*** | NM_012098 | RefSeq | -2.58639 | 0.005732 |
| ***SIPA1L2*** | NM_020808 | RefSeq | -2.599141 | 0.002787 |
| ***SESN2*** | NM_031459 | RefSeq | -2.606985 | 0.000073 |
| ***GOT1*** | NM_002079 | RefSeq | -2.624118 | 0.004033 |
| ***CLDN1*** | NM_021101 | RefSeq | -2.637694 | 0.0033 |
| ***ITGA8*** | NM_003638 | RefSeq | -2.665532 | 0.003202 |
| ***LIF*** | NM_002309 | RefSeq | -2.686526 | 0.004168 |
| ***GREM2*** | NM_022469 | RefSeq | -2.689742 | 0.000464 |
| ***CADM1*** | NM_001098517 | RefSeq | -2.703904 | 0.00231 |
| ***XPOT*** | NM_007235 | RefSeq | -2.706323 | 0.000061 |
| ***FST*** | NM_006350 | RefSeq | -2.718441 | 0.002237 |
| ***ADAMTS5*** | NM_007038 | RefSeq | -2.729646 | 0.00022 |
| ***SLC1A5*** | NM_001145144 | RefSeq | -2.735562 | 0.000917 |
| ***MARS*** | NM_004990 | RefSeq | -2.801902 | 0.000379 |
| ***KRTAP1-5*** | NM_031957 | RefSeq | -2.807386 | 0.011562 |
| ***QSOX1*** | NM_001004128 | RefSeq | -2.879343 | 0.000061 |
| ***SLC3A2*** | NM_001012662 | RefSeq | -2.886606 | 0.001149 |
| ***TM4SF20*** | NM_024795 | RefSeq | -3.046834 | 0.008678 |
| ***SLC38A1*** | NM_001077484 | RefSeq | -3.140269 | 0.000966 |
| ***CDH6*** | NM_004932 | RefSeq | -3.317628 | 0.000098 |
| ***PHGDH*** | NM_006623 | RefSeq | -3.368472 | 0.000049 |
| ***ADAMTS3*** | NM_014243 | RefSeq | -3.442706 | 0.006893 |
| ***SLITRK6*** | NM_032229 | RefSeq | -3.451708 | 0.002212 |
| ***PDE1C*** | NM_001191056 | RefSeq | -3.497536 | 0.000086 |
| ***CHAC1*** | NM_001142776 | RefSeq | -3.58719 | 0.000318 |
| ***COL15A1*** | NM_001855 | RefSeq | -3.620503 | 0.000477 |
| ***WARS*** | NM_004184 | RefSeq | -3.753781 | 0.000049 |
| ***SLC7A11*** | NM_014331 | RefSeq | -3.884104 | 0.001956 |
| ***SEL1L3*** | NM_015187 | RefSeq | -3.954312 | 0.00011 |
| ***DDIT3*** | NM_001195053 | RefSeq | -3.956345 | 0.000134 |
| ***MTHFD2*** | NM_006636 | RefSeq | -4.140162 | 0.000587 |
| ***TGFBI*** | NM_000358 | RefSeq | -4.58175 | 0.012735 |
| ***POSTN*** | NM_001135934 | RefSeq | -4.663144 | 0.001552 |
| ***TNFSF18*** | NM_005092 | RefSeq | -4.703164 | 0.018357 |
| ***FAM129A*** | NM_052966 | RefSeq | -5.173486 | 0.000049 |
| ***SEMA3D*** | NM_152754 | RefSeq | -5.27533 | 0.000061 |
| ***VLDLR*** | NM_001018056 | RefSeq | -5.501593 | 0.000049 |
| ***ASNS*** | NM_001178075 | RefSeq | -6.475575 | 0.000086 |
| ***SLC7A5*** | NM_003486 | RefSeq | -7.892272 | 0.000049 |
| ***VCAM1*** | NM_001078 | RefSeq | -7.983518 | 0.000318 |
| ***PSAT1*** | NM_058179 | RefSeq | -20.800327 | 0.000049 |
