# Supplementary material for: PSAT1 positively regulates the osteogenic lineage differentiation of periodontal ligament stem cells through the ATF4/PSAT1/Akt/GSK3β/β-catenin axis
Source: J Transl Med. 2023 Feb 2;21:70. doi: 10.1186/s12967-022-03775-z (PMC9893676; doi:10.1186/s12967-022-03775-z)
Supplement: Supplementary file 4 — Additional file 4: Figure S1. Validation of microarray results in PDLSCs by qRT-PCR. [file 12967_2022_3775_MOESM4_ESM.docx]

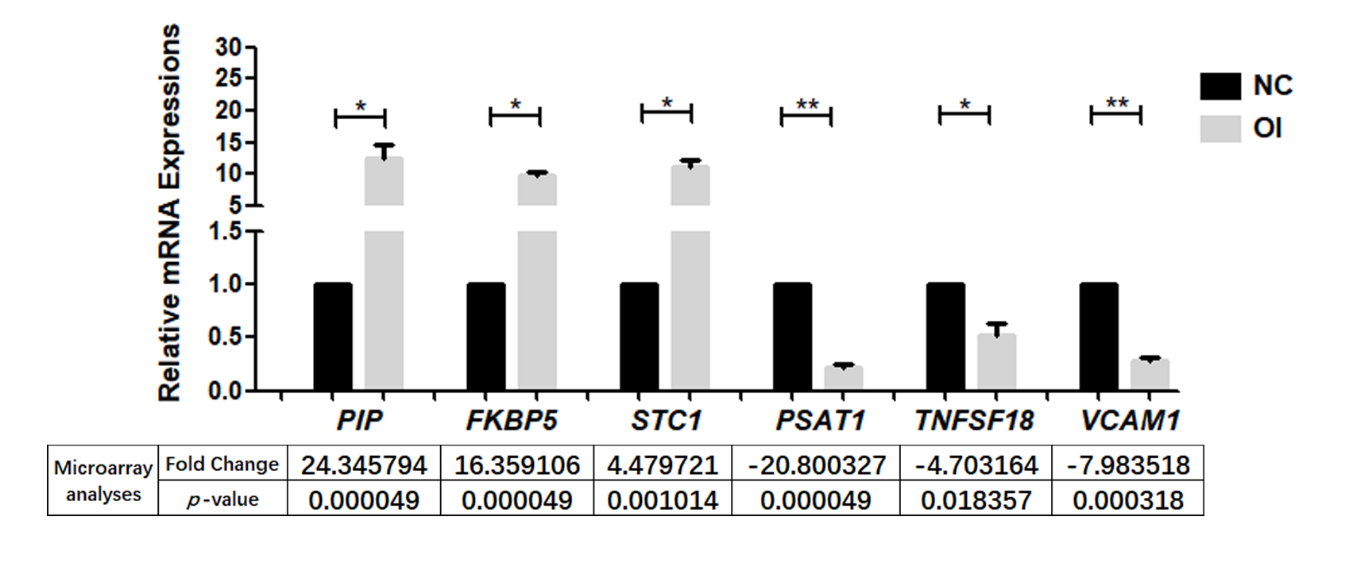


**Figure S1 Validation of microarray results in PDLSCs by qRT-PCR**

To verify the results of microarray assay, the mRNAs levels of 6 genes in PDLSCs after osteogenic induction for 7 days were detected by qRT-PCR. NC: PDLSCs cultured in the complete culture medium for 7 days. OI: PDLSCs cultured in the osteogenic medium for 7 days. *: *p*<0.05. **: *p*<0.01.
