# Supplementary material for: PSAT1 positively regulates the osteogenic lineage differentiation of periodontal ligament stem cells through the ATF4/PSAT1/Akt/GSK3β/β-catenin axis
Source: J Transl Med. 2023 Feb 2;21:70. doi: 10.1186/s12967-022-03775-z (PMC9893676; doi:10.1186/s12967-022-03775-z)
Supplement: Supplementary file 5 — Additional file 5: Figure S2. The interference efficiency of using siRNA to interfere with target genes in PDLSCs. [file 12967_2022_3775_MOESM5_ESM.docx]

**Figure S2 The interference efficiency of using siRNA to interfere with target genes in PDLSCs**

PDLSCs were transfected with siRNAs to inhibit the expression of 8 representative genes with high fold change after osteogenic induction, then the mRNA levels of these genes were detected by qRT-PCR. siNC: PDLSCs transfected with siRNA-targeted none. si-gene: PDLSCs transfected with siRNA-targeted the gene.
