# Supplementary material for: PSAT1 positively regulates the osteogenic lineage differentiation of periodontal ligament stem cells through the ATF4/PSAT1/Akt/GSK3β/β-catenin axis
Source: J Transl Med. 2023 Feb 2;21:70. doi: 10.1186/s12967-022-03775-z (PMC9893676; doi:10.1186/s12967-022-03775-z)
Supplement: Supplementary file 6 — Additional file 6: Table S3. Differentially expressed genes in PDLSCs that have co-expression relationships with PSAT1. [file 12967_2022_3775_MOESM6_ESM.docx]

**Table S3 Differentially expressed genes in PDLSCs that have co-expression relationships with PSAT1**

| **Gene 1** | **Gene 2** | **Correlation Coefficient** | **Relationship** |
| --- | --- | --- | --- |
| ***PSAT1*** | ***RNF41*** | 0.989571 | positive |
| ***PSAT1*** | ***SHMT2*** | 0.997892 | positive |
| ***PSAT1*** | ***TCEA1*** | 0.996734 | positive |
| ***PSAT1*** | ***VLDLR*** | 0.998656 | positive |
| ***PSAT1*** | ***YARS*** | 0.989259 | positive |
| ***PSAT1*** | ***AKAP6*** | 0.991233 | positive |
| ***PSAT1*** | ***ATF4*** | 0.989084 | positive |
| ***PSAT1*** | ***DDIT3*** | 0.99837 | positive |
| ***PSAT1*** | ***GLUL*** | -0.993772 | negative |
| ***PSAT1*** | ***IARS*** | 0.98887 | positive |
| ***PSAT1*** | ***MTHFD2*** | 0.989525 | positive |
